# NCBoost classifies pathogenic non-coding variants in Mendelian diseases through supervised learning on purifying selection signals in humans

**DOI:** 10.1186/s13059-019-1634-2

**Published:** 2019-02-11

**Authors:** Barthélémy Caron, Yufei Luo, Antonio Rausell

**Affiliations:** 1 Clinical Bioinformatics Lab, Imagine Institute, Paris Descartes University, Sorbonne Paris Cité, 75015 Paris, France; 2 INSERM UMR 1163, Institut Imagine, 75015 Paris, France

**Keywords:** Mendelian diseases, Whole genome sequencing, Rare variant analysis, Non-coding genetic variants, Pathogenicity score

## Abstract

State-of-the-art methods assessing pathogenic non-coding variants have mostly been characterized on common disease-associated polymorphisms, yet with modest accuracy and strong positional biases. In this study, we curated 737 high-confidence pathogenic non-coding variants associated with monogenic Mendelian diseases. In addition to interspecies conservation, a comprehensive set of recent and ongoing purifying selection signals in humans is explored, accounting for lineage-specific regulatory elements. Supervised learning using gradient tree boosting on such features achieves a high predictive performance and overcomes positional bias. NCBoost performs consistently across diverse learning and independent testing data sets and outperforms other existing reference methods.

**Electronic supplementary material:**

The online version of this article (10.1186/s13059-019-1634-2) contains supplementary material, which is available to authorized users.

## Background

To date, more than 4000 Mendelian diseases have been clinically recognized [1], collectively affecting more than 25 million people in the USA only [2]. However, around 50% of all known Mendelian diseases still lack the identification of the causal gene or variant [3]. Moreover, every year, approximately 300 new Mendelian diseases are described, whereas the pace for discovery of the causal molecular mechanisms fluctuates at around 200 yearly [3]. Despite the progress achieved through whole exome sequencing (WES)-based studies, recent reviews show highly heterogeneous diagnostic rates across disease types [4, 5], ranging from < 15% (such as congenital diaphragmatic hernia or syndromic congenital heart disease) to > 70% (e.g., ciliary dyskinesia). In those scenarios, a common working hypothesis is that non-coding variants could explain the etiology of many of the unresolved cases [5]. Whole genome sequencing (WGS) allows expanding the survey of pathogenic variants to non-coding genomic regions in an unbiased way. Such possibility generates great expectations, as most trait/disease-associated single-nucleotide variants (SNVs) identified by genome-wide association studies (GWAS) map to non-coding regions, suggesting a prominent role of regulatory elements in genetic diseases [6, 7]. Nevertheless, the large amount of rare and singleton variants in non-exonic positions shown by large-scale WGS projects in humans [8, 9] makes computational predictions a fundamental step to prioritize candidate variants for further clinical and experimental follow-up.

A number of machine learning methods have been developed in the last years to predict the regulatory consequences of non-coding SNVs [10–16]. Two complementary perspectives have been exploited: first, from an evolutionary standpoint, genomic positions under non-neutral evolution are expected to have a functional role. Consequently, position-based purifying selection scores determined at different timescales (i.e., from vertebrates, mammals, and primates sequence alignments) have been successfully used by reference methods. Second, from a mechanistic view, phenotypic consequences of genetic variants are thought to result from their impact on non-coding functional elements, defined as those having reproducible biochemical features associated with regulatory roles, such as promoters, enhancers, silencers, and repressors. Thus, computational methods have exploited diverse sets of chromatin and epigenetic characteristics (including histone marks, chromatin states, DNase I-hypersensitivity sites, and transcription factor binding sites) obtained from heterogeneous sets of cell lines, primary cell types, and tissues by Consortia such as ENCODE, FANTOM5, the Roadmap Epigenomics, and BluePrint projects [17–19]. While the ability of state-of-the-art methods to discriminate functionally relevant non-coding variants is remarkable, the value of such scores as a proxy of pathogenic potential in the context of Mendelian diseases is still unclear. This stems from the fact that functional scores of non-coding SNVs were mostly evaluated by their ability to identify trait-associated (e.g., quantitative trait loci, QTLs) and disease-associated loci from GWAS of common diseases. Yet, even in those contexts, predictive accuracy is modest and mainly driven by position-based interspecies conservation signals, with chromatin and epigenetic features providing only a marginal contribution [11, 14, 16]. More recently, Smedley et al. developed the Regulatory Mendelian Mutation Score (ReMM) [20], which—to our knowledge—is the only method specifically developed to score pathogenic non-coding variants in the context of Mendelian disease studies. Their approach trained a random forest classifier on a curated set of 406 SNVs, including long non-coding RNA SNVs. Twenty-six features were considered, including 8 interspecies conservation scores, 4 GC/CpG-based characteristics, and 8 epigenetic features. Despite the simplicity of the model, ReMM scores proved valuable to prioritize Mendelian disease variants when integrated in a more comprehensive framework considering candidate regulatory regions and the phenotypic relevance of the associated genes [20].

In this work, we hypothesized that the computational prediction of pathogenic non-coding variants in Mendelian diseases would benefit from a more comprehensive set of natural selection signals, notably regarding recent and ongoing selective constraints in humans. In this regard, evolutionary and functional evidence support a rapid turnover of functional non-coding elements across species that would limit the capacity of interspecies conservation to pinpoint recently acquired regulatory sequences in the human lineage [21–24]. Moreover, it has been suggested that lineage-specific and ongoing natural selection in humans could help further understanding the partial overlap observed among the fraction of the genome inferred to be functional from evolutionary, biochemical, and genetic evidences [25, 26]. The use of recent and ongoing purifying selection signals to prioritize pathogenic variants has been historically challenged by a number of confounding factors shaping patterns of human genomic variation. Thus, random genetic drift, population structure, and demographic processes, such as rapid expansions, migrations, and population bottlenecks, have played a major role in governing changes in allele frequency within and between populations [26–28]. In addition, uneven recombination rates across the genome and heterogeneous neutral mutation rates [29, 30] associated with sequence context [31, 32] or to different types of non-coding elements [33] further complicate the distinction of neutral versus non-neutral evolution.

Notwithstanding, the increasing sample size of current large-scale whole genome sequencing projects of the general population is providing a better resolution of recent and ongoing purifying selection signals in humans [9, 34–36], which could improve their utility in scoring systems of pathogenic variants. In addition, machine learning methods have shown ability to detect complex patterns associated with functional variants combining different types of selective constraints that would otherwise be missed by classical approaches [11–16]. A supervised learning approach could thus help in better exploiting recent natural selection features in spite of the confounding factors. To test both previous possibilities, we first extracted a comprehensive set of recent and ongoing natural selection features determined from recent large-scale WGS projects in humans, together with interspecies conservation scores assessed on different evolutionary timescales. We then trained NCBoost, a classifier of non-coding SNVs based on gradient tree boosting, on a curated set of high-confidence pathogenic non-coding SNVs associated with monogenic Mendelian disease genes and on common non-coding SNVs without clinical assertions. The approach outperformed reference state-of-the-art methods under multiple training and testing scenarios, while overcoming gene and positional bias.

## Results

### Curation of a high-confidence set of pathogenic non-coding variants associated with monogenic Mendelian disease genes

**Fig. 1:**
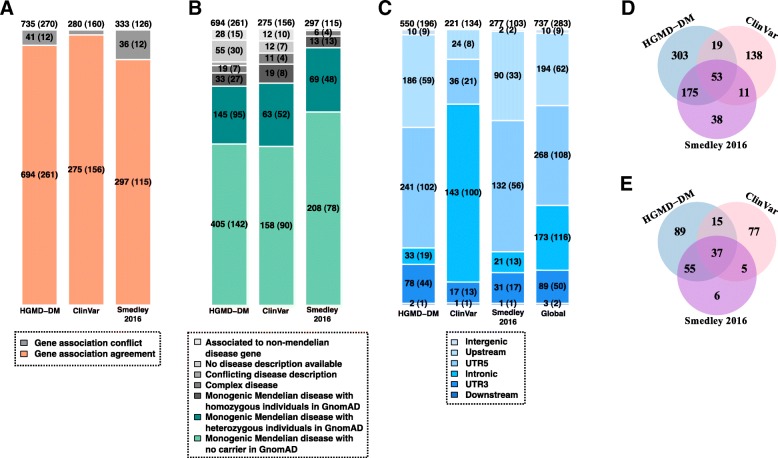
Curation of high-confidence pathogenic non-coding SNVs associated with monogenic Mendelian disease genes. **a** Number of high-confidence pathogenic non-coding SNVs obtained from the Human Gene Mutation Database [37] (HGMD-DM), ClinVar [38], and Smedley’2016 [20], after filtering out SNVs overlapping exonic and splice sites of protein-coding genes and SNVs associated with non-coding RNAs (“Methods” section). Only high-confidence pathogenic non-coding variants associated with the same protein-coding gene by both the original resource and the annotation process done in this work (depicted in orange) were retained for downstream analysis. **b** Retained variants in **a** were further classified according the OMIM category of the associated gene, i.e., non-Mendelian disease gene, Mendelian disease gene associated with a disease phenotype differing from the one reported in the original resource (i.e., presenting a conflicting disease description), complex Mendelian disease genes, and monogenic Mendelian disease genes. Only high-confidence pathogenic non-coding SNVs associated with monogenic Mendelian diseases with no homozygous individuals in GnomAD database [35] (depicted in green) were finally retained for downstream analyses. **c** Distribution of the high-confidence pathogenic non-coding SNVs associated with monogenic Mendelian disease genes according to the type of gene region they overlap: intronic, 5′UTR, 3′UTR, upstream, downstream, and intergenic regions. **d** Distribution of the high-confidence pathogenic non-coding SNVs associated with monogenic Mendelian disease genes according to the original annotation source, i.e., HGMD-DM, ClinVar, and Smedley’2016. **e** Corresponding number of monogenic Mendelian disease genes collectively involved by SNVs in **d**. The number of SNVs in each category is indicated inside the barplots and Venn diagrams together with the number of genes collectively involved (in parenthesis in **a**–**c**; totals are reported above each barplot)

Pathogenic non-coding variants from the Human Gene Mutation Database [37] (HGMD-DM), ClinVar [38], and Smedley’2016 [20] were manually curated to obtain a high-confidence set of pathogenic variants associated to monogenic Mendelian diseases. The number of high-confidence pathogenic non-coding variants obtained from each resource is represented in Fig. 1 (“Methods” section). The majority of causal variants were assigned to the closest protein-coding gene in the reference source (94%, 98%, and 89%, respectively). Thus, the available set is mostly constituted of proximal *cis*-regulatory variants (Fig. 1a), with distal *cis*-regulatory and *trans*-acting variants scarcely represented. Our curation effort allowed further refining this set to retain the fraction of pathogenic variants confidently associated with monogenic Mendelian diseases genes (84%, 87%, and 98%, respectively; Fig. 1b). In addition, a small though non-negligible fraction of variants for which homozygous individuals were detected in recent large-scale whole exome and genome sequencing (GnomAD) were excluded for downstream analysis (5%, 7%, and 4% respectively, Fig. 1b). After all filtering steps, a total of 737 pathogenic non-coding SNVs collectively associated with 283 genes were retained (Fig. 1c; Additional file 1: Table S1). Variants were distributed in intronic (23%), 5′UTR (36%) and 3′UTR (12%), and 1 kb upstream TSS (26%), with a minority of them in 1 kb downstream TSE (< 1%) and in intergenic regions (1%). The three resources mined in this work (HGMD-DM, ClinVar and Smedley’2016) showed varying degrees of overlap regarding causal SNVs (Fig. 1d) and their associated genes (Fig. 1e). Notably, the set of 283 monogenic Mendelian disease genes collectively affected by pathogenic non-coding SNVs is enriched in haploinsufficient genes (odds ratio OR = 2.93, one-sided Fisher test *p* value = 1.279e−09) and in genes intolerant to heterozygous truncation (OR = 1.21; *p* value = 0.041) as compared to a background set of 3354 monogenic Mendelian disease genes (Additional file 2: Figure S1; “Methods” section).

### Distribution of state-of-the-art pathogenicity scores across pathogenic and non-pathogenic SNVs

We explored the distribution of six state-of-the-art pathogenicity scores (CADD, DeepSEA, Eigen, Eigen-PC, FunSeq2, and ReMM; “Methods” section) across the 737 high-confidence non-coding pathogenic variants and 4,960,178 common SNVs without clinical assertions. All evaluated scores showed marked differences depending on the type of gene region involved (i.e., intergenic, intronic, 3′UTR, 5′UTR, upstream, and downstream regions of associated genes; Additional file 2: Figure S2). Thus, the distributions of median scores per gene for pathogenic SNVs in 5′UTR and for SNVs within 1 kb upstream TSS were shifted towards more severe values than those of pathogenic SNVs in 3′UTR, intronic, and intergenic regions. Bias per gene region was also observed across common SNVs without clinical assertions, suggesting that the regulatory region where a variant maps systematically biases the scores (Additional file 2: Figure S2). More strikingly, the distributions of median scores per gene for common SNVs in 5′UTR were not significantly different than those of pathogenic SNVs in 3′UTR and were significantly different (with more severe scores) than those of pathogenic SNVs in intronic regions for the six scores evaluated (two-sided Wilcoxon test *p* values for all pair-wise comparisons are reported in Additional file 3: Table S2). As a corollary, the previous observations warn about (1) the need of matching the relative composition of pathogenic and non-pathogenic SNVs across different gene regions in predictive benchmarks and (2) the relative differences in region distribution across data sets (Fig. 1c) which could compromise the generality of data set-specific benchmarks.

### Ability of natural selection signals to predict pathogenic non-coding SNVs when considered independently

Table 1 summarizes the set of natural selection features extracted for both pathogenic non-coding SNVs and common SNVs without clinical assertions. First, gathered features covered different evolutionary scales and were classified as interspecies natural selection (considering vertebrates, mammals, and primates, excluding human), or recent and ongoing natural selection in humans. Second, features were categorized either as “position-based” when they refer to the specific genomic position where the variant occurred, “window-level” when they refer to a given sequence interval centered in the SNV, or “gene-level” when they refer to the global characteristics of the closest protein-coding gene.

**Table 1:** Natural selection features associated with non-coding single-nucleotide variants mined in this work. Features are classified under different categories depending on the sequence context (i.e., position level, window level, and gene level) and evolutionary scale: interspecies (vertebrates, mammals, and primates, excluding humans), or recent and ongoing natural selection in humans. We note here that the query variant was excluded from the calculations involving mean allele frequencies and mean heterozygosity of a given region and that the variant allele frequency itself was not used as a feature in any training or pathogenicity prediction throughout the study

Category	Sequence context	Evolutionary scale	Model	Feature abbreviation used in this work	Definition	References
Position-level and window-level features	Position-level	Interspecies (mammals)	A	GerpS	Base-wise Rejected Substitution (RS) score defined by Genomic Evolutionary Rate Profiling (GERP++ scores) from mammalian alignments, excluding humans	[63]
		Interspecies (mammals)	A	GerpN	Neutral evolution score defined by GERP++, excluding humans	[63]
		Recent and ongoing in humans	B	bStatistic	b Statistic: background selection score indicating the expected fraction of neutral diversity that is present at a site, with values close to 0 representing near complete removal of diversity as a result of selection and values near 1 indicating little effect. B-statistic was based on human single-nucleotide polymorphism data from Perlegen Sciences, HapMap phase II, the SeattleSNPs NHLBI Program for Genomic Applications, and the NIEHS Environmental Genome Project	[20]
		Interspecies (primates)	A	priPhCons	Primate PhastCons conservation score, excluding humans	[42, 43]
		Interspecies (mammals)	A	mamPhCons	Mammalian PhastCons conservation scores, excluding humans	[13, 14]
		Interspecies (vertebrates)	A	verPhCons	Vertebrate PhastCons conservation score, excluding humans	[42, 43]
		Interspecies (primates)	A	priPhyloP	Primate PhyloP conservation score, excluding humans	[44]
		Interspecies (mammals)	A	mamPhyloP	Mammalian PhyloP conservation score, excluding humans	[44]
		Interspecies (vertebrates)	A	verPhyloP	Vertebrate PhyloP conservation score, excluding humans	[44]
	1000-bp window	Recent and ongoing in humans	B	meanDaf1000G	Mean derived allele frequency of variants in 1-kb window region calculated from the 1000 Genomes Project (excluding the query variant)	[11]
		Recent and ongoing in humans	B	meanHet1000G	Mean heterozygosity of 1-kb window region calculated from the 1000 Genomes Project (excluding the query variant)	[11]
		Recent and ongoing in humans	B	meanMAF1000G	Mean minor allele frequency of variants in 1-kb flanking region calculated from 1000 Genomes Project (excluding the query variant)	[35]
		Recent and ongoing in humans	B	meanMAFGnomAD meanMAF_AFRGnomAD meanMAF_AMRGnomAD meanMAF_EASGnomAD meanMAF_FINGnomAD meanMAF_NFEGnomAD meanMAF_OTHGnomAD meanMAF_ASJGnomAD	Mean minor allele frequency of variants in 1-kb window region calculated from GnomAD genome data (excluding the query variant from the calculation). Mean MAF was assessed for the global population and for population-specific frequencies: Africans and African Americans (AFR), Admixed Americans (AMR), East Asians (EAS), Finnish (FIN), non-Finnish Europeans (NFE), Ashkenazi Jewish (ASJ), and other populations (OTH)	[35]
	30-kb window	Recent and ongoing in humans	B	TajimasD_CHB_pvalue TajimasD_CEU_pvalue TajimasD_YRI_pvalue	Tajima’s D *p* value: neutrality test that compares estimates of the number of segregating sites and the mean pair-wise difference between sequences. The test is performed within 3 subpopulations of the 1000 Genome Project, producing population-specific scores.	[65]
	30-kb window	Recent and ongoing in humans	B	FuLisD_CEU_pvalue FuLisD_CHB_pvalue FuLisD_YRI_pvalue FuLisF_CEU_pvalue FuLisF_CHB_pvalue FuLisF_YRI_pvalue	Fu and Li’s F* *p* value: neutrality test that compares the number of singletons with the average number of nucleotide differences between pairs of sequences. Fu and Li’s D* *p* value: neutrality test that compares the number of singletons with the total number of mutations in a genomic region within a group. These tests are performed within 3 subpopulations of the 1000 Genome Project, producing population-specific scores.	[35]
	10-bp window	Recent and ongoing in humans	B	CDTS	The Context-Dependent Tolerance Score (CDTS) represents the difference between observed and expected variations in Humans. The expected variation is computed for each nucleotide genome-wide as the probability of variation of each nucleotide depending on its heptanucleotide context. CDTS was computed on 11,257 unrelated individuals.	[36]
	75-bp flanking region	*N/A*	D	GC	Percent GC in a window of ± 75 bp	[63]
		*N/A*	D	CpG	Percent CpG in a window of ± 75 bp	[63]
Gene-level features	Non-coding region of the closest gene (a)	Recent and ongoing in humans	C	ncRVIS	Non-coding RVIS is a measure of the departure from the genome-wide average number of common variants found in the non-coding sequence of genes with a similar amount of non-coding mutational burden in humans. ncRVIS was computed on an in-house collection of whole genome sequencing of 690 individuals.	[72]
		Interspecies (mammals)	C	ncGERP	Average GERP++ score across a gene’s non-coding sequence	[46]
	Coding region of the closest gene	Interspecies (primates)	C	dN/dS	Primate dN/dS ratio, providing a measure of the coding-sequence conservation across primates	[68]
		Recent and ongoing in humans	C	pLI	Probability of being loss-of-function intolerant (intolerant of heterozygous and homozygous loss-of-function variants), assessed from the ExAC database.	[35]
		Recent and ongoing in humans	C	RVIS percentile	Residual Variation Intolerance Score (RVIS) percentile, a measure of the departure from the average number of common functional mutations in genes with a similar amount of mutational burden in humans. RVIS was assessed on sequence data from 6503 whole exome from the NHLBI Exome Sequencing Project (ESP)	[71]
		Recent and ongoing in humans	C	GDI	Gene Damage Index, a gene-level metric of the mutational damage that has accumulated in the general population, based on CADD scores and on the 1000 Genomes Project data	[70]
	Phylo-genetic gene features	*N/A*	C	familyMemberCount	Number of human paralogs of the gene: Family member count (FMC) in OGEE database	[74]
		*N/A*	C	gene_age	The gene age is estimating the origination time of genes from the presence or absence of orthologs in the vertebrate phylogeny.	[73]

*bp* base pairs, *GERP* Genomic Evolutionary Rate Profiling, *RS* Rejected Substitution, *N/A* not applicable

(a) Non-coding region of the closes gene defined in the original publication of ncRVIS and ncGERP as the collection of 5′UTR, 3′UTR, and an additional non-exonic 250 bp upstream of transcription start site (TSS)

For the purpose of this work, features were grouped under three main sets (Table 1): (A) interspecies sequence conservation features at position and window level, (B) recent and ongoing natural selection signals in humans at position and window level, and (C) gene-based features. Furthermore, we included two additional sets of features: (D) the sequence context, i.e., GC and CpG content as well as information of the type of gene region where SNVs overlap (intronic, 5′UTR, 3′UTR, upstream, downstream, and intergenic region); and (E) epigenetic features such as histone modification marks, nucleosome position, open chromatin profiles, and transcription factor binding site (TFBS) profiles generated by the ENCODE project [17].

**Fig. 2:**
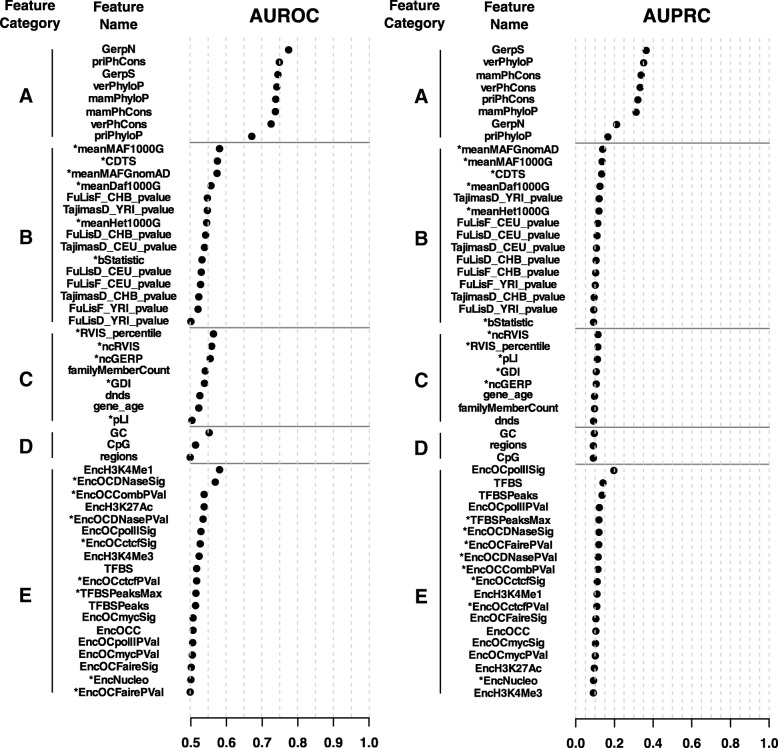
Performance of individual features mined in this work to classify high-confident set of *n* = 737 pathogenic non-coding SNVs associated with monogenic Mendelian disease genes from a negative set of *n* = 7370 randomly sampled common SNVs without clinical assertions matched by region. The area under the receiver operating characteristic curve (AUROC; left panel) and the area under the precision-recall curve (AUPRC; right panel) obtained for each feature is represented. Features are gathered according to five categories (A–E; “Methods” section) and ranked within category by decreasing AUROC and AUPRC. AUROC values < 0.5 (anti-classifiers) were transformed in 1-AUROC values for the purpose of this figure and are indicated with an asterisk (*). Accordingly, AUPRC values for anti-classifiers were assessed on the basis of the − 1 product transformation. Of note, population-specific GnomAD MAFs (“Methods” section) are not shown for simplicity. One hot-encoded SNV region features (i.e., “intronic,” “UTR5,” “UTR3,” “upstream,” “downstream,” and “intergenic”) are gathered as a single feature labeled as “region”

We first investigated the predictive ability of each individual feature to classify the high-confidence set of 737 pathogenic non-coding SNVs associated with monogenic Mendelian disease genes from a “negative set” of 7370 randomly sampled common SNVs without clinical assertions and matched by region (Additional file 4: Table S3; “Methods” section). Figure 2 shows the area under the receiver operating characteristic curve (AUROC) and the area under the precision-recall curve (AUPRC) obtained for each feature. A random classification would be characterized by AUROC values close to 0.5 and by AUPRC values close to 0.1 (corresponding to the fraction of “positive” SNVs among the total SNVs considered). On the other extreme, a perfect classification would show an AUROC = 1 and an AUPRC = 1. The ranking of features according to both AUROC and AUPRC showed that predictive ability was dominated by interspecies sequence conservation features at position and window level (category A), while only poor performances were observed for the rest of the features when considered independently (AUROC < 0.6 and AUPRC < 0.2).

### Supervised learning based on a comprehensive set of ancient, recent, and ongoing purifying selection signals in humans: the NCBoost method

Supervised learning was performed in this work using XGBoost [39, 40], a machine learning approach based on gradient tree boosting (GTB; “Methods” section). GTB performs predictions based on an ensemble of regression trees. Unlike random forest, where the component trees are trained independently, in GTB, trees are built in a stepwise manner, where each successive tree is optimized on the residuals of the prediction of the preceding tree. XGBoost was trained on a “positive set” of 283 high-confident set of pathogenic non-coding SNVs associated with monogenic Mendelian disease genes (randomly selecting one variant per gene out of the total 737 initially obtained to avoid gene-level contamination of the training/testing sets; Fig. 1c) and a negative set of 2830 randomly sampled common SNVs without clinical assertions, matched by region and allowing a maximum of one negative variant per gene (“Methods” section).

The method implemented in this work, called NCBoost, is thus a bundle of 10 independently trained XGBoost models, consecutively excluding in each of them 1 out of 10 genome partitions where “positive” and “negative” variants are evenly distributed. In such a way, each non-coding variant in a putative *cis*-regulatory region of a protein-coding gene can be scored in NCBoost by the model that excluded from its training all variants—either pathogenic or non-pathogenic—associated with the same gene. This strategy permits to reduce overfitting as well as to avoid biasing the score of newly seen variants by the fact that they mapped in the vicinity of variants and genes initially presented to the classifier. Therefore, NCBoost may be applied to score any set of non-coding variants in *cis*-regulatory regions with no contamination with the training set. Interestingly, the 10 models proved to be largely equivalent among them, as shown by the high correlation of their scores when applied to an independent set of variants excluded from their training (average Spearman correlation 0.96 ± 0.0111 of all pair-wise comparisons among the 10 models; “Methods” section).

**Fig. 3:**
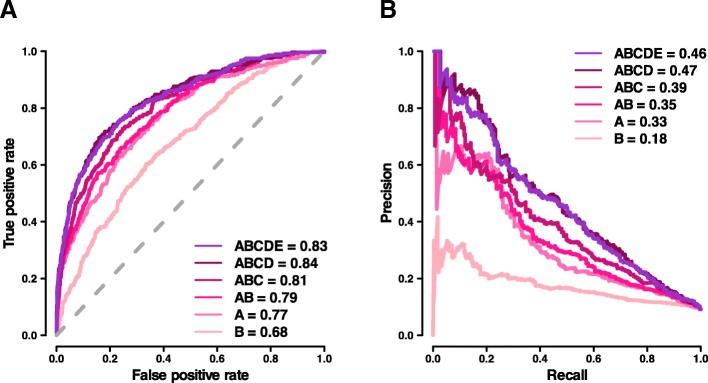
Comparative performance of NCBoost models trained upon different sets of features. The figure represents the area under the receiver operating characteristic curve (AUROC; **a**) and the area under the precision-recall curve (AUPRC; **b**) obtained for each of the six feature configurations evaluated (feature categories A, B, A+B, A+B+C, A+B+C+D, and A+B+C+D+E) when tested mimicking a tenfold cross-validation on *n* = 283 high-confidence pathogenic non-coding SNVs and *n* = 2830 common variants without clinical assertions

Six feature configurations were evaluated, including the following combinations of feature categories: A, B, A+B, A+B+C, A+B+C+D, and A+B+C+D+E (Table 1; “Methods” section). The different NCBoost configurations were first tested mimicking a tenfold cross-validation on the same 283 high-confidence pathogenic non-coding SNVs and 2830 common variants. Figure 3 shows the receiver operating characteristic curve (ROC) and the precision-recall curve (PRC) obtained for each of the six feature configurations. In these figures, a perfect prediction would be represented by the (0,1) corner in ROC space (AUROC = 1) and the (1,1) corner in PRC space (AUPRC = 1). In our case, best performance was reached by the model including ABCD features: AUROC_ABCD_ = 0.84 and AUPRC_ABCD_ = 0.47. The figures represent a relative improvement of 9% (AUROC) and 42% (AUPRC) over a model based purely in interspecies sequence conservation features at position and window level. Results were consistent when NCBoost was trained and tested on positive variants from each of the three resources taken independently, i.e., HGMD-DM, ClinVar, and Smedley’2016 (Additional file 2: Figure S3A, S3B and S3C, respectively). Of note, ABCD and ABCDE models showed a similar performance in terms of AUROC and AUPRC across the different settings evaluated (Fig. 3 and Additional file 2: Figure S3A, S3B and S3C).

The feature importance analysis of NCBoost revealed a balanced contribution of interspecies sequence conservation features at position and window level (feature category A, cumulative importance CI_A|ABCD_ = 42% in the ABCD configuration) and recent and ongoing natural selection signals in humans at position and window level collectively considered (category B, CI_B|ABCD_ = 33%; Additional file 2: Figure S4A and S4B). Such balance is observed in spite of the sharp differences in predictive ability observed across features when considered independently (Fig. 2). The collective feature importance of recent and ongoing natural selection signals in humans is in turn much higher than what could be expected from the observed incremental performance obtained in the joint model AB (interspecies and intraspecies selection) as compared to A (interspecies selection; Fig. 3). Both previous observations, however, are not merely a straightforward consequence of the correlation structure across features (Additional file 2: Figure S5). These results show the capacity of a supervised learning approach using regression trees to extract complex patterns of natural selection signals distinguishing pathogenic versus non-pathogenic non-coding variants.

Notably, in contrast with the state-of-the-art methods evaluated (Additional file 2: Figure S2), the per-region distribution of NCBoost scores showed a clearer separation between high-confidence non-coding pathogenic variants and common SNVs for all types of regions evaluated (Additional file 2: Figure S6). Thus, the distribution of median scores per gene for common SNVs in 5′UTR was significantly lower (i.e., less severe) than that of pathogenic SNVs in all evaluated regions (i.e., intronic, 3′UTR, 5′UTR, and upstream regions; two-sided Wilcoxon test *p* values < 1e−10; Additional file 3: Table S2), with the exception of intergenic region, where low sample size undermined statistical power (Fig. 1c).

### Comparative benchmark against state-of-the-art methods

**Fig. 4:**
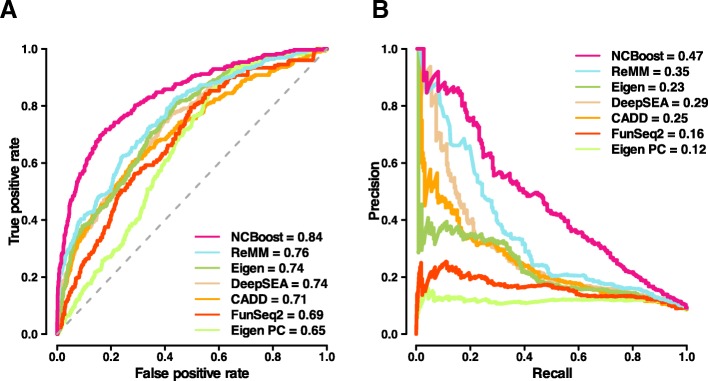
Comparative performance of NCBoost against state-of-the-art methods. The figure shows the area under AUROC (**a**) and the AUPRC (**b**) obtained for NCBoost (configuration of features ABCD) together with the six state-of-the-art methods (CADD, DeepSEA, Eigen, Eigen-PC, FunSeq2, and ReMM; “Methods” section) when tested on the same set of positive and negative variants described for Fig. 3

The NCBoost performance observed in Fig. 3 (configuration ABCD) was compared against the results of the six state-of-the-art methods considered in this work (CADD, DeepSEA, Eigen, Eigen-PC, FunSeq2, and ReMM) when applied on the same positive and negative set of SNVs (Fig. 4). NCBoost outperformed all evaluated methods both regarding AUROC and AUPRC, with a relative improvement of 10% and 34% respectively over the second ranked method (ReMM) and of 13% and 104% over the third ranked method (Eigen). Figures were consistent when the benchmarking was performed on positive variants from each of the three resources taken independently, i.e., HGMD-DM, ClinVar, and Smedley’2016 (Additional file 2: Figure S7A, S7B and S7C, respectively).

The comparative benchmark was then focused on scoring methods that—like NCBoost—are based on supervised learning on non-coding pathogenic variants that are highly enriched in Mendelian diseases. To that aim, we considered (i) GWAVA, trained on HGMD variants [11]; (ii) the more recent ncER score, trained both on HGMD-DM and ClinVar variants [41]; and (iii) ReMM, trained on Smedley’2016 variants [20]. Contrary to NCBoost and ReMM scores, in which a given SNV is never scored by a model that used it for learning, GWAVA and ncER methods could not be included in the benchmark represented in Fig. 4 because of the large overlap of their training set with the pathogenic variants evaluated therein. To overcome this issue, we performed a dedicated comparative benchmark for GWAVA and ncER on the non-overlapping part of their training set with the high-confidence pathogenic SNVs curated in this work. NCBoost outperformed reference methods in both settings (Additional file 2: Figure S8).

We then assessed the ability of the different methods to discriminate pathogenic variants seemingly unconstrained across mammalian evolution. Thus, we selected 234 of the 737 high-confidence pathogenic variants from the initial set that presented PhastCons [42, 43] and PhyloP [44] scores for mammals which were below the median value of the 7370 common variants sampled for training (0.001 and − 0.014, respectively). As expected, the performance of all evaluated methods decreased for this subset of pathogenic variants (Additional file 2: Figure S9). Despite the more challenging scenario, NCBoost still remained as the best performing method, with relative improvements of 17% and 57% in AUROC and AUPC over the second best ranked method (Eigen PC). NCBoost’s superior performance to discriminate pathogenic variants that are seemingly unconstrained across mammalian evolution highlights its ability to exploit more recent signals of purifying selection, both in primates and in humans.

The outperformance of NCBoost over reference methods was also observed when testing on the same positive set of 283 high-confident set of pathogenic non-coding SNVs as in Fig. 4 and on a negative set that—rather than of common variants—is composed of 2830 randomly selected rare variants (allele frequency < 1%) matched by region (Additional file 2: Figure S10; “Methods” section). This test allows ruling out the possibility that figures obtained in Fig. 4 are merely explained by the capacity to discriminate rare from common variants, rather than to discriminate pathogenic from non-pathogenic variants.

In a more stringent setup, we further explored the capacity of the different methods to discriminate pathogenic and non-pathogenic variants within the same non-coding region of a given gene. For this purpose, we restricted the previous test to a set of 149 region-matched pairs of pathogenic and random common variants associated with 54 unique genes (Additional file 2: Figure S11). Results obtained were consistent with those previously observed in Fig. 4 and in Additional file 2: Figure S10, further supporting the superior ability of NCBoost to discriminate pathogenic variants as compared to reference methods. We note that in Additional file 2: Figures S8, S9, S10, S11 no re-training of NCBoost was done, but the same NCBoost _ABCD_ bundle trained as described in the previous section was applied.

### Independent training and testing across all possible configurations of the three sources of high-confidence non-coding pathogenic SNVs

To characterize the performance of the NCBoost approach upon different training and testing scenarios, we evaluated all possible configurations of the training and testing sets upon the three sources of high-confidence non-coding pathogenic SNVs, i.e., HGMD-DM, ClinVar, and Smedley’2016. Thus, the positive set of 283 high-confident set of pathogenic non-coding SNVs and the associated negative set of 2830 common SNVs matched by region (“Methods” section) were each split in two non-overlapping sets in three different ways according to the annotation source (Table 2). Accordingly, we trained on the pathogenic variants reported in one source and tested on those in the other two sources not overlapping with the first one. In addition, we explored two additional configurations: training on variants reported in at least two sources and testing on those reported only in one single source, and vice versa. For each different training set, we retrained NCBoost as a bundle of 10 independently trained models, consecutively excluding in each of them 1 out of 10 genome partitions as previously done for the entire sets. We note again that a maximum of one positive and one negative variant per gene was allowed within the positive and negative sets used for training. In addition, in all previous training/testing configurations, we further required that there was no overlap between the genes associated with SNV variants in the training set and those of the independent testing set, regardless of their positive and negative classification. Table 2 shows the AUROC and AUPRC values obtained on each of the independent testing sets when applying the corresponding NCBoost model. Consistent with previous results, NCBoost outperformed the reference state-of-the-art methods under all training and testing evaluated scenario.

**Table 2:** AUROC and AUPRC values obtained by NCBoost upon different configurations of the training and independent testing sets. The figures obtained by the six state-of-the-art methods evaluated on the same testing sets are shown together with NCBoost

Positive set used for NCBoost training		Positive set used for testing		AUROC							AUPRC						
Source	# SNVs	Source	# SNVs	CADD	DeepSEA	Eigen	Eigen PC	FunSeq2	ReMM	NCBoost	CADD	DeepSEA	Eigen	Eigen PC	FunSeq2	ReMM	NCBoost
HGMD-DM	186	!HGMD-DM	97	0.67	0.76	0.78	0.70	0.67	0.74	0.82	0.16	0.24	0.23	0.13	0.15	0.26	0.36
CV	107	!CV	176	0.72	0.74	0.73	0.63	0.68	0.77	0.78	0.27	0.31	0.24	0.11	0.15	0.37	0.38
Smedley	78	!Smedley	205	0.69	0.72	0.73	0.65	0.66	0.75	0.78	0.24	0.25	0.21	0.11	0.14	0.31	0.36
≥ 2 sources	73	1 source	210	0.68	0.72	0.73	0.64	0.66	0.75	0.78	0.23	0.24	0.21	0.11	0.14	0.31	0.31
1 source	210	≥ 2 sources	73	0.8	0.81	0.82	0.69	0.77	0.82	0.85	0.31	0.43	0.29	0.16	0.24	0.48	0.52

### Validation on a set of recently discovered non-coding pathogenic variants

**Fig. 5:**
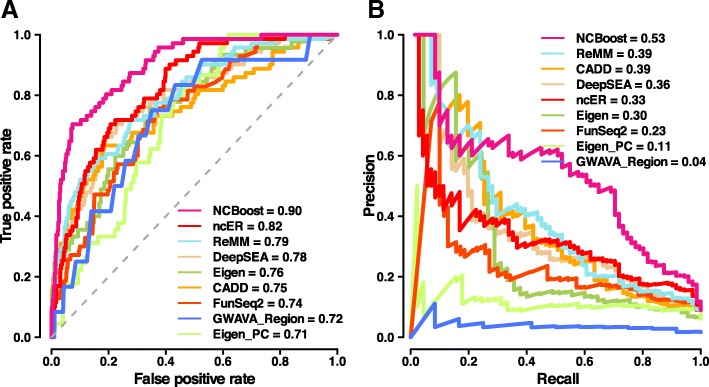
Testing of NCBoost against a fully independent set of recently reported pathogenic variants. The figure shows the area under AUROC (**a**) and the AUPRC (**b**) obtained for NCBoost (configuration of features ABCD) together with the eight reference methods (CADD, DeepSEA, Eigen, Eigen-PC, FunSeq2, ReMM, GWAVA, and ncER; “Methods” section) tested on a fully independent set of 70 positive and 700 negative variants matched per genomic region (“Methods” section). Only the GWAVA Region version is depicted for the sake of visualization. GWAVA Unmatched and TSS versions led to AUROC of 0.59 and 0.58 and to AUPRC of 0.02 and 0.02, respectively. Notice that GWAVA and ncEM methods were considered here assuming no overlap of their training sets with the recently reported pathogenic non-coding variants evaluated in these figures. There was no re-training of NCBoost, but the same NCBoost ABCD bundle used in Figs. 3 and 4 was applied

We evaluated NCBoost’s performance in a fully independent validation set of non-coding pathogenic variants. For this, we retrieved 70 SNVs in non-coding regions of protein-coding genes newly reported in recent updates of the HGMD and ClinVar databases. The new pathogenic SNVs collectively associated with 47 genes and involve a total of 45 human diseases (details provided in Additional file 5: Table S4; “Methods” section). For the purpose of this analysis, each pathogenic SNV was associated with a unique set of ten randomly sampled negative common human variants, matched to the positive set to maintain the same fraction of variants per type of region (Additional file 5: Table S4). Figure 5 shows the predictive ability of NCBoost in such validation test in terms of AUROC and AUPRC values. As in previous settings, NCBoost outperformed reference methods, including GWAVA and ncER. Importantly, NCBoost values on the validation set (AUROC = 0.90 and AUPRC = 0.53; Fig. 5) were similar to those observed in the independent test performed by mimicking a tenfold cross-validation (AUROC = 0.84 and AUPR = 0.47; Fig. 4). The consistency of values observed between the test and validation datasets supports the lack of overfitting in the training process.

### Prioritization of non-coding pathogenic variants within individual genomes

**Fig. 6:**
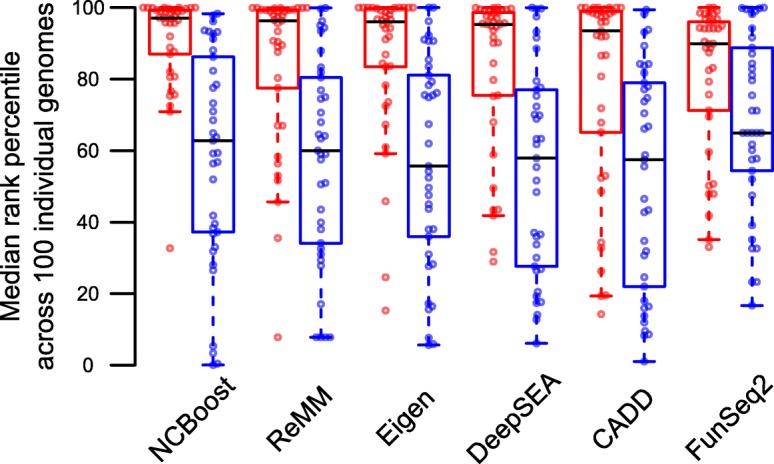
Prioritization of non-coding pathogenic variants within individual genomes. The median across the 100 simulated disease genomes of the within-individual rank percentile of a variant (*y*-axis) is shown for 37 recently reported pathogenic variants evaluated (red dots) and their corresponding 37 randomly sampled negative common SNVs mapping within the same non-coding region of the associated genes (blue dots). Boxplots represent the distributions for the different evaluated scores (*x*-axis). NCBoost scores offered the highest within-individual rank percentiles of pathogenic variants (median 97.04%), with a statistical significant difference (one-sided paired Wilcoxon test *p* value) as compared to all evaluated reference methods: ReMM (median 96.34%; *p* value = 3.75e−2), Eigen (median 96.00%, *p* value = 9.55e−2), DeepSEA (95.3%; *p* value = 1.96e−3), CADD (median 93.50; *p* value = 4.03e−3), FunSeq2 (median 89.92; *p* value = 5.88e−4). All scores showed a statistically significant difference between the median rank percentile distribution of pathogenic and their internal control negative variants (one-sided paired Wilcoxon test): NCBoost *p* value = 8.04E-07, ReMM *p* value = 9.71E−05, Eigen *p* value = 2.28E−05, DeepSEA *p* value = 7.51E−06, CADD *p* value = 3.88E−04, and FunSeq2 *p* value = 6.13E−03. Complete details are provided in Additional file 6: Table S5. An alternative graphical representation pairing each pathogenic variant with is provided in Additional file 2: Figure S12

In a more realistic setting for the study of monogenic Mendelian diseases, the efficacy of scoring methods should be given by their ability to prioritize the causal pathogenic non-coding variant among the variants found in a patient’s genome. To mimic such scenario, we simulated disease genomes by adding a specific pathogenic variant to the variants found in healthy individuals from the 1000 Genomes Project [8]. For this purpose, we restricted the analysis to the 37 pathogenic variants in the validation set that mapped to autosomal chromosomes (to avoid a gender effect of the background genomes) and that could be associated with a randomly sampled negative common SNV mapping within the same non-coding region of the affected gene. Such negative variants are intended to serve here as internal negative controls, accounting thus for eventual biases both at the level of the region type (Additional file 2: Figure S2) and of the affected gene. Each pathogenic variant and its associated “negative variant” were independently spiked in 100 randomly sampled individual genomes. Their scores were ranked against those of non-coding variants in proximal *cis*-regulatory regions per individual (median per individual of 1,031,931 SNVs, collectively affecting a median of 16′378 protein-coding genes per individual; “Methods” section). Median values across the 100 simulated disease genomes of the within-individual rank percentile are indicated in Fig. 6 and Additional file 2: Figure S12 for each of the 37 pathogenic variants independently evaluated and for their corresponding 37 negative variants used as internal controls. NCBoost scores provided the highest within-individual rank percentiles of pathogenic variants (median 97.04%), with a statistical significant difference as compared to all reference methods evaluated (one-sided paired Wilcoxon test *p* value < 0.05), with the exception of Eigen for which a *p* value = 0.095 was obtained. No statistical differences were observed among the NCBoost scores of the negative control variants and those of the evaluated methods (one-sided paired Wilcoxon test *p* value > 0.05 in all cases), permitting to exclude systematic biases. Notably, all scores showed a statistically significant difference between the median rank percentile distribution of pathogenic and their corresponding internal control negative variants. However, NCBoost’s score leads to the strongest separation of pathogenic and internal control variants, as reflected by the one-sided paired Wilcoxon test with *p* value = 8.04e−7. Exact *p* values of the previous tests are reported in Fig. 6 legend, and complete details per variant are provided in Additional file 6: Table S5. GWAVA and ncER scores were not evaluated in this section because of the intrinsic bias of their publicly available genome-wide scores as a consequence of the contamination with the corresponding training set. Thus, the class labels presented for training the classifier will bias the genome-wide scoring of variants in the vicinity of such training set.

### Case study: NCAPD3 intron variant NC_000011.9:g.134086816T>C associated with autosomal recessive primary microcephaly-22

Here, we present the variant NC_000011.9:g.134086816T>C, a recently reported pathogenic SNV to illustrate the added value of assessing recent and ongoing purifying selection signals in humans through a supervised machine learning approach. This is an intronic variant of the NCAPD3 gene (non-SMC condensin II complex subunit D3) and was found in compound heterozygous state in combination with the frameshift mutation NC_000011.9:g.134063952del in a patient with autosomal recessive primary microcephaly-22 (MCPH22 [45]). Mutations were detected by whole exome sequencing, confirmed by Sanger sequencing, and segregated with the disease in the family. In the patient’s fibroblasts, the NC_000011.9:g.134086816T>C mutation leads to skipping exon 3 which, in combination with the frameshift variant in trans, resulted in markedly reduced NCAPD3 protein expression. Low levels of normally spliced transcript and wild-type protein were nevertheless detected. Thus, the mutation caused a decrease in NCAPD3 function but was not considered functionally null [1, 45]. This variant was poorly scored by all reference methods evaluated, with a median of within-individual rank percentiles ranging between 90.69% (ReMM) and 35.11% (FunSeq2) (Additional file 2: Figure S12). In contrast, NCBoost scored the g.134086816T>C variant as highly pathogenic, i.e., in the top 99.26% percentile (Additional file 7: Table S6). Detailed inspection of the purifying selection features associated to this SNV showed that the affected genomic position is weakly conserved across vertebrates and mammals and mildly conserved across primates (Additional file 7: Table S6). When considered globally, however, the non-coding sequence of the NCAPD3 gene is constrained, as reflected by high non-coding GERP values [46]. Interestingly, complementary genomic position features suggest high levels of recent and ongoing purifying selection in humans, as reflected by low mean derived allele frequency, mean heterozygosity, Tajima’s D and Fu and Li’s F* and D* values (Additional file 7: Table S6). Despite the fact that none of the features are conclusive when considered independently, NCBoost’s model managed to incorporate the complex pattern they convey and assign a high pathogenicity score.

### Scoring of non-coding genomic regions associated with monogenic Mendelian disease genes

**Fig. 7:**
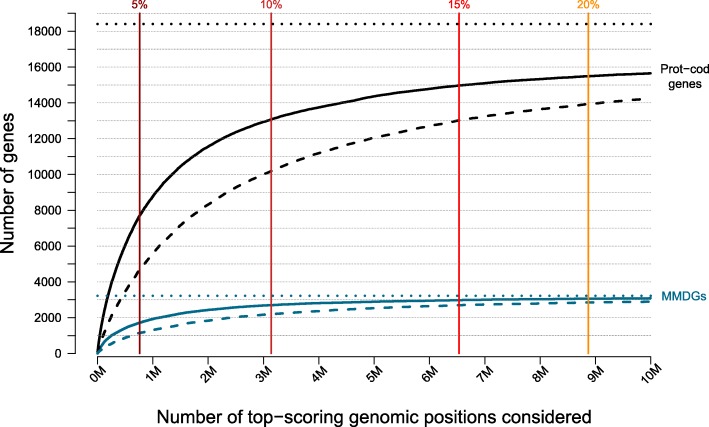
The figure represents the number of monogenic Mendelian disease genes (MMDGs, in blue, *y*-axis) bearing at least one (solid curves) or ten (dashed curves) potentially pathogenic non-coding variants as a function of the top NCBoost scoring positions considered (*x*-axis), ranked from left (more pathogenic) to right (less pathogenic). A total of 857,825,085 positions overlapping intronic, 5′UTR or 3′UTR, and upstream and downstream regions collectively associated with 18,404 protein-coding genes was used as a reference background. For the sake of visualization, the *x*-axis was cut at 10 Million top-scoring genomic positions. Vertical bars represent the thresholds at which left positions display NCBoost scores higher than the corresponding top percentage of the high-confidence pathogenic variants curated in this work. Top 5%, 10%, 15%, and 20% thresholds are represented. The horizontal dotted lines represent the total of 3223 MMDGs (in blue) and 18,404 protein-coding genes (in black) for which NCBoost scores were obtained. Genes bearing potentially pathogenic non-coding variant per gene within the top 5% and the top 10% are highly enriched in MMDGs as compared to the background of protein-coding genes used (see text)

We applied NCBoost to 189,829,714 genomic positions overlapping intronic, 5′UTR or 3′UTR, upstream and downstream regions associated with 3223 monogenic Mendelian disease protein-coding genes (MMDGs) for which annotations were retrieved (“Methods” section). Among them, a total of 261,507 and 980,219 genomic positions presented an NCBoost score higher than the top 5% and the top 10%, respectively, of the high-confidence pathogenic variants curated in this work. These values translate in a total of 1715 and 2699 MMDGs having at least one potentially pathogenic non-coding variant per gene with an NCBoost score higher than the top 5% and the top 10% of the high-confidence pathogenic variants, respectively (Fig. 7). These results represent a significant enrichment of MMDGs among the protein-coding genes bearing at least one highly pathogenic variants, with gene-based odds ratio OR_Top_5%_ = 1.27, Fisher’s test *p* value_Top_5%_ = 3.78e−13; and OR_Top_10%_ = 1.18, *p* value_Top_10%_ = 8.81e−09, respectively, based on a reference background of 18,404 protein-coding genes collectively associated with 857,825,085 non-coding positions. In a more stringent setting, a total of 1146 and 2199 MMDGs were found with at least ten potentially pathogenic non-coding variants per gene with an NCBoost score higher than the top 5% and the top 10%, respectively, of the reference set of high-confidence pathogenic variants. Here, the corresponding gene-based enrichments increase with OR_Top_5%_ = 1.4 and Fisher’s test *p* value_Top_5%_ = 6.2e−18 and OR_Top_10%_ = 1.23 and *p* value_Top_10%_ = 6.48e−12. Besides the 283 MMDG currently showing high-confidence pathogenic non-coding variants (as curated in this work, Fig. 1), our estimates suggest that an additional number of 1040 MMDGs have the potential to cause a disease on the basis of highly pathogenic SNVs in proximal *cis*-regulatory regions.

## Discussion

In this study, we implemented a supervised learning approach, called NCBoost, to classify pathogenic SNVs based on a comprehensive set of features at the position, flanking region, and gene level, associated with interspecies, recent and ongoing selection in humans. When trained and tested on multiple configurations of high-confidence sets of pathogenic non-coding SNVs associated with monogenic Mendelian disease genes, this approach showed superior performance than the reference methods. Notable improvements were observed on precision-recall rates. The context-specific assessment of natural selection signals permitted to overcome the pervasive regional bias observed in all evaluated reference methods, which, e.g., tend to provide scores to non-pathogenic common SNVs in 5′UTR not significantly different from the scores assigned to high-confident pathogenic SNVs in 3′UTR and significantly different (with more severe scores) than those of pathogenic SNVs in intronic regions.

The rigorous curation process showed that current sets of high-confidence large-effect pathogenic non-coding SNVs associated with monogenic Mendelian diseases are mostly constituted of proximal *cis*-regulatory variants associated with the closest protein-coding gene, in line with previous reports [20]. Such distribution most probably reflects a historical ascertainment bias towards such regions in previously described Mendelian genes, which is expected to be steadily overcome by unbiased WGS approaches [5]. However, for the time being, the current status poses limits to the supervised learning and benchmarking on distal *cis-* and *trans-*acting pathogenic regulatory variants with clinical implications in Mendelian diseases. Additionally, it warns about the applicability and expected performance of our approach and reference state-of-the-art methods for such scope. On the other extreme, the use of common variants as a surrogate of non-pathogenic variants for the supervised training process cannot rule out their potential contribution to disease susceptibility and modifier effects, mostly in the context of polygenic diseases and complex traits. In the scope of monogenic Mendelian diseases addressed by this work, however, such limitation is expected to have a minor impact.

The implementation of NCBoost allowed us to evaluate the ability to prioritize pathogenic non-coding SNVs of recent and ongoing natural selection features in humans when considered independently, collectively, and in combination with interspecies conservation. While none of the evaluated features showed individual predictive strength (Fig. 2), supervised learning performed through gradient tree boosting found complex patterns associated with pathogenic SNVs, reaching a significant performance combining multiple features (Fig. 3). Detailed feature importance analysis showed a prominent contribution of recent and ongoing natural selection signals under all evaluated feature configurations. However, their final impact in the global performance of the classifier, while remarkable, is attenuated by the fact that some signals may be redundant with selective constrains already accounted for by interspecies conservation. The best results were, nonetheless, obtained when the collective assessment of interspecies and intraspecies natural selection features was performed taking into consideration the sequence context where SNVs occurred, as informed by the selective signals accumulated by the associated gene and by the type of non-coding element involved.

This work represents a proof-of-concept of the added value of incorporating a large and heterogeneous set of recent and ongoing natural selection features in humans under a supervised machine learning approach for the detection of pathogenic non-coding SNVs associated with Mendelian diseases. The rapidly increasing sample size of current large-scale WGS projects in the general population is expected to have a major impact in the capacity to detect additional and more accurate recent and ongoing natural selection signals in humans, with a consequent repercussion in their use to identify pathogenic non-coding variants, as recently illustrated [36, 47, 48].

In the last years, different large-scale projects have identified an important fraction of regulatory elements of the human genome, and the epigenetic insights are proving valuable to understand the functional consequences of disease-associated variants in those regions [17–19]. However, in the setting of this work, the small set of evaluated epigenetic features had only a minor contribution to the classification of pathogenic SNVs associated with Mendelian diseases, in line with the results of previous analyses [11, 14, 16]. On the one hand, this may suggest that the epigenetic signals evaluated here are partially redundant with natural selection features; a more exhaustive extraction of epigenetic features was however beyond the scope of this work. On the other hand, it may reflect a lack of specificity in regard to the cell types and tissues relevant for the heterogeneous set of Mendelian diseases considered here. In this line, recent studies are consolidating a view of regulatory mechanisms that is highly cell type-specific, where gene expression, DNA methylation, histone modifications, promoter interaction networks, and transcription factor binding sites may substantially vary across tissues and developmental stages [49–52]. Thus, the assessment of non-coding variants in the context of Mendelian diseases may largely benefit from the integration of purifying selection signals with the epigenetic information derived on the particular cell types, tissues, and/or developmental time relevant for the onset and progression of a disease, as illustrated by recent successful examples [48, 53]. Recently, a computational approach based on a latent Dirichlet allocation model modeling data from multiple cell types and tissues (FUN-LDA) was shown to predict functional impact of non-coding variants in a cell type- and tissue-specific manner [54]. Notwithstanding, the identification of the specific cell type and tissue to be considered may be a challenging task, especially in the case of largely uncharacterized rare Mendelian diseases and syndromes with pleiotropic clinical signs.

Recently, it was shown that the number of singleton variants found on each newly sequenced genome stabilizes on average at ~ 8,500, with regulatory elements highly enriched in the relative amount of SNVs found per kilobase of sequence [9]. The large amount of rare variants in each individual genome, together with the typically low number of participants in the study of specific rare diseases, challenges the statistical power of downstream statistical association and/or linkage studies to relate a genotype with a phenotype. The scoring approaches evaluated in this work may help filtering variants to increase power, although they often need to be integrated within more comprehensive frameworks in order to reach the necessary sensitivity and specificity to identify causal variants in disease cohorts [20]. In addition to the use of epigenetic information previously discussed, variant filtering strategies include focusing on SNVs associated with genes of phenotypic relevance for the disease under consideration [20, 55]. From a complementary perspective, gene-based or region-based aggregation tests of multiple variants (a class of rare variant association tests) have been developed to evaluate cumulative effects of multiple genetic variants in a gene or region. The aim of such aggregation strategies is to increase the statistical power when multiple variants may be associated with a disease [56], e.g., in the case of burden tests and variance component tests implemented in popular software such as PLINK/SEQ, SEQSpark, and SKAT. In these approaches, a continuous weight function can be used in the aggregation of rare variants in order to up-weight those predicted to have more damaging consequences. A similar weighting strategy can be proposed for rare variant extensions of the transmission disequilibrium test in the analysis of parent-child trio data [57]. In both previous families of statistical tests for rare variant analysis of WGS from Mendelian disease studies, the pathogenic scores led by the supervised learning approach implemented in this work, NCBoost, may be used to weight the aggregation of candidate pathogenic SNVs across heterogeneous *cis*-regulatory elements in a consistent way overcoming position and region biases.

## Conclusions

Current large-scale WGS projects on the general population are increasingly providing the necessary sample size to detect recent and ongoing purifying selection signals in humans. The results obtained in this work shows that, when integrated in a supervised learning framework, the assessment of a comprehensive set of such signals improves the prediction of large-effect pathogenic non-coding variants associated to Mendelian diseases. Through the evaluation of interspecies and human-specific natural selection features at the affected position, the flanking region, and the associated gene, the NCBoost method outperformed reference methods under multiple scenarios. Notably, the scores produced by this supervised approach overcome positional bias, thus permitting a consistent weighting and aggregation of candidate variants across diverse non-coding regions for downstream statistical analyses.

## Methods

### High-confidence pathogenic variants

Three sets of high-confidence pathogenic variants in non-coding regions were obtained: (1) regulatory disease-causing mutations (“DM” set) from the Human Gene Mutation Database (HGMD, professional version, accessed on 2018/01/03 [37]), manually annotated as involved in conferring the associated clinical phenotype; (2) pathogenic SNVs from ClinVar [38] manually annotated as “pathogenic” with no conflicting assertions (GrCh37 release from [58]); and (3) a manually curated set compiled from the medical literature of non-coding SNVs associated with Mendelian diseases and validated by experimentation or co-segregation studies, or for which other convincing evidence of pathogenicity was available.

### Variant mapping and annotation of non-coding SNVs

Only single-nucleotide variants (SNVs) where considered through the study. Variants were annotated using ANNOVAR software [59] (downloaded from [60]) using the gene-based annotation option based on RefSeq (assembly version hg19) in order to obtain (i) the gene region affected by intragenic variants or (ii) the nearest flanking gene in the case of intergenic variants. Exonic variants and variants within 10 base pairs (bp) of a splicing junction of protein-coding genes were removed (ANNOVAR *splicing_threshold* = 10). At this stage, variants from HGMD-DM, ClinVar, and Smedley’2016 overlapping non-coding RNAs within an exon (*n* = 143, 2, and 68, respectively), intron (*n* = 24, 3, and 13, respectively), or 10 bp from a splicing junction (*n* = 1, 0, and 0, respectively) were filtered out. In the case of SNVs overlapping several types of regions associated with different genes or transcripts, the following three criteria were consecutively adopted: (A) the default ANNOVAR precedence rule for gene-based annotation was applied, i.e., exonic = splicing > ncRNA > UTR5 = UTR3 > intronic > upstream = downstream > intergenic. (B) if after the previous step a SNV could still be associated with several neighbor/overlapping genes (e.g., in the intergenic region between two genes, or in the intronic region of two overlapping genes, etc.), the SNV’s nearest protein-coding gene was kept as a reference for the annotation of the variant. The SNV’s nearest gene was determined by the shortest distance to either the TSS or TSE. (C) Pathogenic SNVs with two or more genes with identical shortest distance to TSS/TSE were tagged as “conflicting” and filtered out from the analysis. After all previous filtering steps, a total of 18 disease-causing SNVs overlapping upstream (*n* = 9), 3′UTR (*n* = 7), and downstream regions (*n* = 2) of non-coding RNAs were filtered out. Thus, for the purpose of this study, the set of non-coding variants was constituted of SNVs associated with protein-coding genes and overlapping intronic, 5′UTR or 3′UTR, upstream and downstream regions—i.e., closer than 1 kb from the transcription start site (TSS) and the transcription end site (TSE) respectively—and intergenic regions.

### Curation of high-confidence pathogenic non-coding SNVs associated with monogenic Mendelian disease genes

Among the set of high-confidence pathogenic SNVs, we manually supervised a total of 71 cases showing a disagreement between the gene associated with the variant in the original resource (i.e., HGMD-DM, ClinVar, and Smedley’2016) and the gene associated by the previously described annotation procedure. The original gene assignment was kept for 17 SNVs where conflict originated due to straightforward exceptions of the ANNOVAR’s precedence rule or the assignment to the nearest upstream or downstream gene (criteria A and B described in the previous section). The number of variants retained at this stage is represented in Fig. 1a. Only high-confidence pathogenic non-coding variants associated with the same gene by both the original resource and the annotation process done in this work were retained for downstream analyses.

We then evaluated whether the genes associated with high-confidence pathogenic non-coding SNVs were reported as Mendelian disease genes in OMIM [1]. A list of 3695 Mendelian disease genes was obtained following Chong et al. [3]: OMIM raw data files were downloaded from [61]. Phenotype descriptions containing the word “somatic” were flagged as “somatic,” and those containing “risk,” “quantitative trait locus,” “QTL,” “{,” “[,” or “susceptibility to” were flagged as “complex.” Mendelian genes were then defined as those having a supporting evidence level of 3 (i.e., the molecular basis of the disease is known) and not having a “somatic” flag. Two main categories of Mendelian disease genes were defined: monogenic Mendelian disease genes (*n* = 3354) and complex Mendelian disease genes (*n* = 596), i.e., those presenting mutation risk factors and quantitative trait loci (QTL) or contributing to susceptibility to multifactorial disorders or to susceptibility to infection [3]. Two hundred fifty-five genes were associated with both monogenic and complex Mendelian disease genes.

High-confidence pathogenic non-coding SNVs associated with monogenic Mendelian disease genes were further inspected manually to check consistency between the disease phenotype reported in the original source (i.e., HGMD-DM, ClinVar, and Smedley’2016) and the ones described in OMIM database for the same gene. A total number of 138 variants for which the agreement was unclear or non-existent were filtered out for downstream analyses. In the remaining set of pathogenic non-coding SNVs, we then inspected whether variants were detected as heterozygous or homozygous among the individuals included in the GnomAD database [35] (version r2.0.2), using both WES and WGS data. Variants present as homozygous in at least one carrier were filtered out for downstream analysis. Thus, only high-confidence pathogenic non-coding SNVs associated with monogenic Mendelian diseases, with no homozygous individuals in GnomAD and overlapping intronic, 5′UTR, 3′UTR, upstream, downstream, and intergenic regions, were finally retained for downstream analysis (Additional file 1: Table S1).

### Common and rare human variants without clinical assertions

Common and rare human variants without clinical assertions where obtained from dbSNP [62]. Variants labeled as common (“COMMON = 1”) and with minor allele frequency (MAF) > 0.05 were considered as “common variants,” while those labeled as non-common (“COMMON = 0”) and with MAF < 0.01 were annotated as “rare variants.” Variants with no MAF information (no “CAF” field reported in the “INFO” field of the variant) as well as multi-allelic variants were filtered out. Common and rare human variants without clinical assertions were first annotated by ANNOVAR and filtered as described above. For consistency in the comparison against the pathogenic set of SNVs, the set of common and rare human variants without clinical assertions was restricted to SNVs associated with protein-coding genes and overlapping intronic, 5′UTR, 3′UTR, upstream, downstream, and intergenic regions. The list of protein-coding genes was extracted from Ensembl Biomart [63] (human genome assembly version GrCh37.p13).

### Pathogenicity scores of non-coding SNVs

Pre-computed pathogenicity scores of non-coding SNVs were extracted from the following state-of-the-art methods: CADD non-coding score (version v1.3 [10]), DeepSEA functional significance score (version v0.94 [14], a -log10 transformation was used throughout this work), Eigen and Eigen-PC scores (version v1.1 [16]), FunSeq2 score (version v1.2 [12]), ReMM scores (version v0.3.1 [20]), GWAVA scores (version 1,0 [11]), and ncER scores (ncER_10bpBins_percentile_version1 created on August 13, 2018 [41]).

### Feature extraction of non-coding SNVs

Extracted features are summarized in Table 1 and can be classified in five main categories.

#### Interspecies sequence conservation features at position and window level

To evaluate evolutionary conservation at a given site, the following scores evaluating non-neutral rates of substitution from multiple species alignments (excluding humans) were used: PhastCons [42, 43] and PhyloP [44] scores for three multi-species alignment (vertebrates, mammals, and primates) and GerpN and GerpS single-nucleotide scores from mammalian alignments [64], all of them obtained from CADD (version v1.3 [10]). PhyloP scores measure neutral evolution at individual sites. The score corresponds to the -log *p* value of the null hypothesis of neutral evolution. Positive values (up to 3) represent purifying selection, while negative values (up to − 14) represent acceleration. PhastCons scores estimate the probability that the locus is contained in a conserved element. GerpN and GerpS single-nucleotide scores assess respectively the neutral substitution rate and the rejected substitution rate of the locus. A high GerpN value indicates high homology of the locus across species. Positive values of GerpS indicate a deficit in substitutions, while negative values convey a substitution surplus.

#### Recent and ongoing natural selection signals in humans at position and window level

Three human population-specific natural selection scores based on the allele frequency spectrum on a 30-kb sequence window region centered around the SNV were obtained from The 1000 Genomes Selection Browser 1.0 [65]: Tajima’s D [66], Fu and Li’s D*, and Fu and Li’s F* [67]. Tajima’s D is a neutrality test comparing estimates of the number of segregating sites and the mean pair-wise difference between sequences. Fu and Li’s D* is a neutrality test comparing the number of singletons with the total number of nucleotide variants within a region. Fu and Li’s F* is a neutrality test comparing the number of singletons with the average number of nucleotide differences between pairs of sequences. The three tests were performed within three populations of the 1000 Genome Project phase 1 data, producing population-specific scores [65]: Yoruba in Ibadan, Nigeria (YRI); Han Chinese in Beijing, China (CHB); and Utah Residents with Northern and Western European Ancestry (CEU). Negative logarithmic percentiles associated with each of these scores were used with values ranging from 0 (indicating positive selection) to 6 (indicating purifying selection).

The background selection score (B statistic [68]), assessing the expected fraction of neutral diversity that is present at a site, was obtained from CADD annotations (version v1.3). B statistic values close to 0 represent nearly complete removal of diversity as a result of selection whereas values near 1 indicate no conservation. The B statistic is based on human single-nucleotide polymorphisms from Perlegen Sciences, HapMap phase II, the SeattleSNPs NHLBI Program for Genomic Applications, and the NIEHS Environmental Genome Project.

Context-dependent tolerance score (CDTS) for 10-bp bins of the human genome computed on 11,257 unrelated individuals was obtained from [36]. CDTS represents the difference between observed and expected variations in humans. The expected variation is computed genome-wide for each nucleotide as the probability of variation of each nucleotide based on its heptanucleotide context. Low CDTS indicates loci that are intolerant to variation.

Mean heterozygosity and mean derived allele frequency of variants in a 1-kb window region centered on the SNV and calculated from the 1000 Genomes Project (excluding the query variant) were obtained from GWAVA v1.0 source data [69]. Mean minor allele frequency (MAF) of variants in a 1-kb window were calculated from GnomAD genome data [35], excluding the query variant from the calculation. Mean MAF was assessed for the global population and for population-specific frequencies: Africans and African Americans (AFR), Admixed Americans (AMR), East Asians (EAS), Finnish (FIN), non-Finnish Europeans (NFE), Ashkenazi Jewish (ASJ), and other populations (OTH). Additionally, we extracted mean MAF of variants in a 1-kb window calculated from the 1000 Genomes Project (excluding the query variant). MAFs both from GnomAD and the 1000 Genomes Project were extracted from GnomAD release r2.0.2.

#### Gene-based features

The following gene-level features associated with natural selection were obtained:

Primate dn/ds ratios (i.e., the ratio between the number of nonsynonymous substitutions and the number of synonymous substitutions) were taken from [70]. Low dn/ds values reflect purifying selection, while high dn/ds values are indicative of positive selection.

The gene probability of loss-of-function intolerance (pLI [35]), estimating the depletion of rare and de novo protein-truncating variants as compared to the expectations drawn from a neutral model of de novo variation on ExAC exomes data, was obtained from the ExAC Browser (release 0.3.1 [71]). pLI values close to 1 represent gene intolerance to heterozygous and homozygous loss-of-function mutations.

Gene damage index (GDI), a gene-level metric of the mutational damage that has accumulated in the general population, based on CADD scores, was taken from [72]. High GDI values reflect highly damaged genes.

The Residual Variation Intolerance Score (RVIS percentile, provided in [73]) assesses the gene departure from the average number of common functional mutations in genes with a similar amount of mutational burden in humans. High RVIS percentiles reflect genes which are highly tolerant to variation.

The non-coding version of the RVIS score (ncRVIS, as calculated in [46]) measures the departure from the genome-wide average of the number of common variants found in the non-coding sequence of genes with a similar amount of non-coding mutational burden in humans. Negative values of ncRVIS indicate a conserved proximal non-coding region, while positive values indicate a higher burden of SNVs than expected under neutrality.

The average non-coding GERP (ncGERP) is the average GERP score [64] across a gene’s non-coding sequence [46]. Both in the case of ncRVIS and ncGERP, the non-coding sequence was defined in the original publication as the collection of 5′UTR, 3′UTR, and an additional non-exonic 250 bp upstream of the transcription start site (TSS).

Gene age, estimating the gene time of origin based on the presence/absence of orthologs in the vertebrate phylogeny, was taken from [74]. It varies from 0 (oldest) to 12 (youngest, corresponding to human-specific genes). The number of human paralogs for each gene was collected from the OGEE database [75].

For all scores, gene names were mapped to approved gene symbols from HUGO Gene Nomenclature Committee (HGNC [76]). Missing values were imputed through the median value computed over all protein-coding genes.

#### Sequence context

The percentage of GC and CpG in a window of 150 bp around the variant of interest was taken from CADD v1.3 annotations. In addition, we obtained the non-coding genomic region overlapping the SNV using ANNOVAR and encode it as binary features: intronic, 5′UTR, 3′UTR, upstream, downstream, and intergenic regions.

#### Epigenetic features

Epigenetic features such as histone modification marks, nucleosome position, open chromatin profiles, and transcription factor binding site (TFBS) profiles generated by the ENCODE project [17] were extracted from CADD v1.3 annotations.

DNA accessibility was assessed using the following sets of features: (1) the open chromatin evidence coming from the open chromatin super track, containing peak signal and Phred-scaled *p* values of evidence for five open chromatin assays: DNase-seq (EncOCDNaseSig and EncOCDNasePVal), FAIRE-seq (EncOCFaireSig and EncOCFairePVal), and ChIP-seq using CTCF (EncOCctcfSig and EncOCctcfPVal) and PolII (EncOCpolIISig and EncOCpolIIPVal) and Myc (EncOCmycSig and EncOCmycPVal); (2) The Phred-scaled combined *p* value of both DNase-seq and FAIRE-seq assays (EncOCCombPVal); (3) The Open Chromatin Code (EncOCC), a metric integrating DNaseI, FAIRE, and ChIP-seq peak evidence of open chromatin; and (4) the maximum nucleosome position score obtained through MNase-seq (EncNucleo), indicating packed chromatin states.

Potential transcription factor activity was assessed using (i) the number of different overlapping TFBS (TFBS), (ii) the number of overlapping TFBS peaks summed over cell types (TFBSPeaks), and (iii) the highest value of the overlapping TFBS peaks across cell types from ChIP-seq (TFBSPeaksMax). In addition, the following histone modification marks were used: the maximum methylation peak at H3K4 (EncH3K4Me1, enhancers-associated), maximum trimethylation peak at H3K4 (EncH3K4Me3, promoter-associated), and maximum acetylation peak at H3K27 (EncH3K27Ac, associated with active enhancers).

### NCBoost training strategy

NCBoost training was performed with XGBoost, a machine learning technique based on gradient tree boosting (also known as gradient boosted regression tree [39, 40]). XGBoost R implementation (version 0.71.1) from [39] was used with parameters: eta = 0.01, max_depth = 25, and gamma = 10, selected to avoid overfitting and after parameter optimization through a prior tenfold cross-validation step. All features evaluated (Table 1) are quantitative or binary and were presented to XGBoost without normalization or standardization. To train NCBoost, we first randomly split the complete list of protein-coding genes in 10 genome partitions of equal size, with the same distribution across all chromosomes and keeping in each of them the same proportion of monogenic Mendelian disease genes presenting high-confidence pathogenic non-coding variants (see above). Throughout the work, each pathogenic SNV (that is, a positive variant) was associated with a unique set of 10 negative variants, randomly sampled from the set of common human variants without clinical assertion described above and associated with genes within the same genome partition. Random sampling of common variants was matched to the positive set to keep the same fraction of variants per type of region: intronic, 5′UTR, 3′UTR, upstream, downstream, and intergenic regions. A maximum of one positive and one negative variant associated with the same gene was allowed, although no minimum per gene was required. For the training step, a maximum of one pathogenic non-coding variant was randomly sampled per gene (Additional file 4: Table S3). We then trained NCBoost as a bundle of 10 independently trained XGBoost models, consecutively excluding in each of them 1 of the 10 genome partitions described above. We note here that each SNV received one single score—and not 10 different scores—which was provided by the model that did not contain the genome partition where the SNV overlapped.

### Correlation between independently trained 10 XGBoost models within the NCBoost bundle

To assess the correlation among the scores led by the independently trained 10 XGBoost models, we established 11 genome partitions in order to create 11 independent sets of positive and negative variants, randomly sampled in an analogous way as described above. One partition was randomly selected and reserved for validation while the other 10 were used for training. Ten XGBoost models were then independently trained using the set of features A+B+C+D described above, by consecutively excluding in each of them 1 of the 10 genome partitions. Then, each model was used to score variants in the 11th partition. In this specific context and only for the purpose of this evaluation, each SNVs overlapping with the 11th partition received 10 different scores generated by the 10 models, respectively. Correlation among the scores of the 10 models was assessed through Spearman rank correlation.

### Random sampling of rare human variants without clinical assertion

Each disease-causing variant was associated with a unique set of 10 rare variants, randomly sampled from the set of rare human variants without clinical assertion described above. Random sampling of rare variants was matched to the positive set to keep the same fraction of variants per type of region: intronic, 5′UTR, 3′UTR, upstream, downstream, and intergenic regions. A maximum of one positive and one rare variant associated with the same gene was allowed, although no minimum per gene was required.

### Region-based random sampling of common variants

To constitute a “region-context” matched set of positive and negative variants, each disease-causing variant was associated—when available—with one common variant, randomly sampled from the set of common human variants without clinical assertion associated with the same gene and mapping to the same region (intronic, 5′UTR, 3′UTR, upstream, downstream, and intergenic regions). Disease-causing variants with no matching common variants in the same region of the same gene were excluded from the region-context matched set of positive and negative variants. Multiple positive-negative variant pairs per gene were allowed in this setting. A total of 149 region-matched pairs of pathogenic and random common variants, collectively, associated with 54 unique genes, were sampled.

### Independent set of newly discovered non-coding pathogenic variants

Recently reported pathogenic variants in non-coding regions were obtained from (1) Regulatory disease-causing mutations (“DM” set) from the Human Gene Mutation Database (HGMD, professional version, accessed on 2018/10/08 and not previously reported on 2018/01/03 [37]) and (2) pathogenic SNVs from ClinVar [38] manually annotated as “pathogenic” with no conflicting assertions (GrCh37 release from 2018/09/30 not previously reported in [58]). Variant mapping and annotation of non-coding SNVs was performed as described above. No further curation was performed on this set.

### Simulated disease genomes

One hundred individuals with population code EUR were randomly sampled from the 1000 Genome Project Phase 3 (2013/05/02 release [8]). Individual codes were HG01708, HG00311, HG01524, HG01509, HG01784, NA20516, NA12546, NA12414, NA20581, HG00251, HG00181, HG01707, NA20803, NA20507, HG02238, HG00373, HG01686, HG01631, HG00281, NA11831, HG01702, HG00186, NA20525, HG01334, HG00145, NA11832, NA12282, NA20799, HG00123, HG00245, NA12234, HG00246, HG01624, NA20502, NA20514, NA20509, HG00351, NA20754, HG00269, HG01762, NA20588, HG00100, NA20762, HG01608, NA20585, HG01527, HG01618, HG01704, NA20538, NA12813, NA12400, NA20811, HG00255, HG00338, NA20512, HG00148, NA10847, HG02223, NA20800, HG02221, HG00320, NA20755, HG01537, HG00097, HG00182, HG00239, HG00371, HG00258, HG02236, NA12154, HG00242, HG00304, HG00315, HG00331, HG01679, HG01528, NA12751, NA10851, HG00357, HG00325, HG01670, NA20806, HG00106, HG00368, HG00237, HG00238, NA20826, NA12890, HG00263, HG00120, NA20770, NA07051, HG00278, NA20535, NA12761, NA07056, HG01531, HG01771, HG00250, and NA12716. Multi-allelic loci were expanded and treated as independent variants. Variant mapping, annotation, and filtering of non-coding SNVs were performed as described in the corresponding section above. In order to analyze simulated disease genomes, the set of non-coding variants was restricted to SNVs associated with protein-coding genes and overlapping intronic, 5′UTR or 3′UTR, and upstream and downstream regions as previously defined. Standard competition ranking was used to estimate within-individual rank percentiles.

### Annotation of haploinsufficient genes

A list of 299 haploinsufficient genes was obtained from [77]. Genes intolerant to heterozygous truncation (pLI > 0.9 [35]) were obtained from ExAC Browser (release 0.3.1 [71]; file fordist_cleaned_exac_nonTCGA_z_pli_rec_null_data.txt).

## Additional files

Additional file 1: **Table S1.** Subset of the 737 curated disease-causing non-coding variants. (XLSX 53 kb)

Additional file 2: Supplementary Figure 1 to Supplementary Figure 12. (PDF 3207 kb)

Additional file 3: **Table S2.** Regional bias of pathogenic score distributions. (XLSX 11 kb)

Additional file 4: **Table S3.** Random common variants matched by region to the pathogenic variants from Table S2. (XLSX 291 kb)

Additional file 5: **Table S4.** Validation set including a “positive” set of 70 SNVs in non-coding regions of protein-coding genes newly reported in recent updates of the HGMD and ClinVar databases, and a “negative” set of 700 randomly sampled common human variants, matched per type of region to the “positive” set. (XLSX 36 kb)

Additional file 6: **Table S5.** Prioritization of 37 non-coding pathogenic variants and their associated 37 internal control negative common variants spiked in within 100 individual genomes. (XLSX 17 kb)

Additional file 7: **Table S6.** Feature and pathogenicity scores for intron variant 11:134086816.T > C associated with gene NCAPD3. (XLSX 12 kb)

## Abbreviations

AUPRC: Area under the precision-recall curve
AUROC: Area under the receiver operating characteristic curve
GTB: Gradient tree boosting
GWAS: Genome-wide association study
SNVs: Single-nucleotide variants
TFBS: Transcription factor binding site
WES: Whole exome sequencing
WGS: Whole genome sequencing
