# Supplementary material for: NCBoost classifies pathogenic non-coding variants in Mendelian diseases through supervised learning on purifying selection signals in humans
Source: Genome Biol. 2019 Feb 11;20:32. doi: 10.1186/s13059-019-1634-2 (PMC6371618; doi:10.1186/s13059-019-1634-2)
Supplement: Supplementary file 2 — Supplementary Figure 1 to Supplementary Figure 12. (PDF 3207 kb) [file 13059_2019_1634_MOESM2_ESM.pdf]

Supplementary Figures

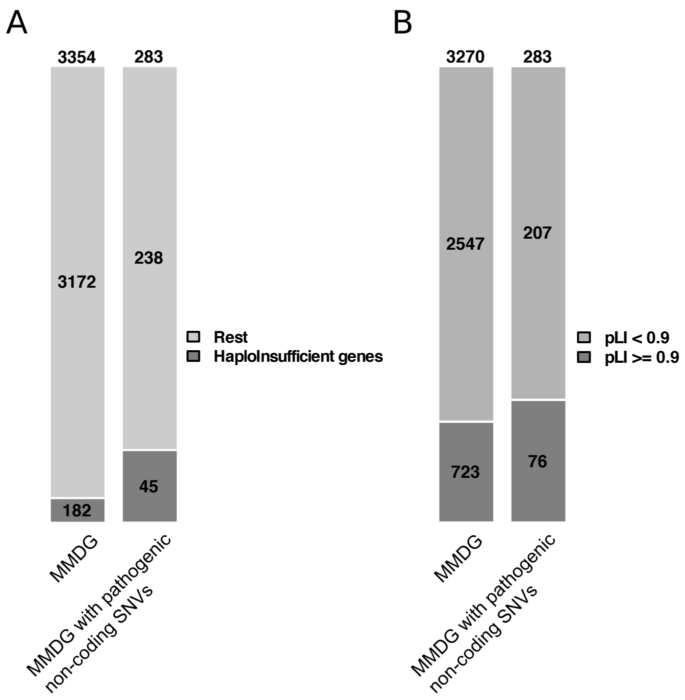

Figure S1. Relative enrichment of monogenic Mendelian disease genes associated with high-confidence pathogenic non-coding SNVs in haploinsufficient and dominant genes.
Barplots showing the distribution of all monogenic Mendelian disease genes (n=3354; abbreviated as MMDG for the purpose of this figure) and those MMDG associated with high-confidence pathogenic non-coding SNVs (n=283) among the following categories: **A**. Haploinsufficient genes from (1). **B.** Genes intolerant to heterozygous truncation (pLI>0.9, (2)). One-sided Fisher test p-values assessing the enrichment of monogenic Mendelian disease genes associated with high-confidence pathogenic non-coding SNVs in haploinsufficient and dominant genes were: 1.279e-09 (Panel A), and 0.04107 (Panel B)

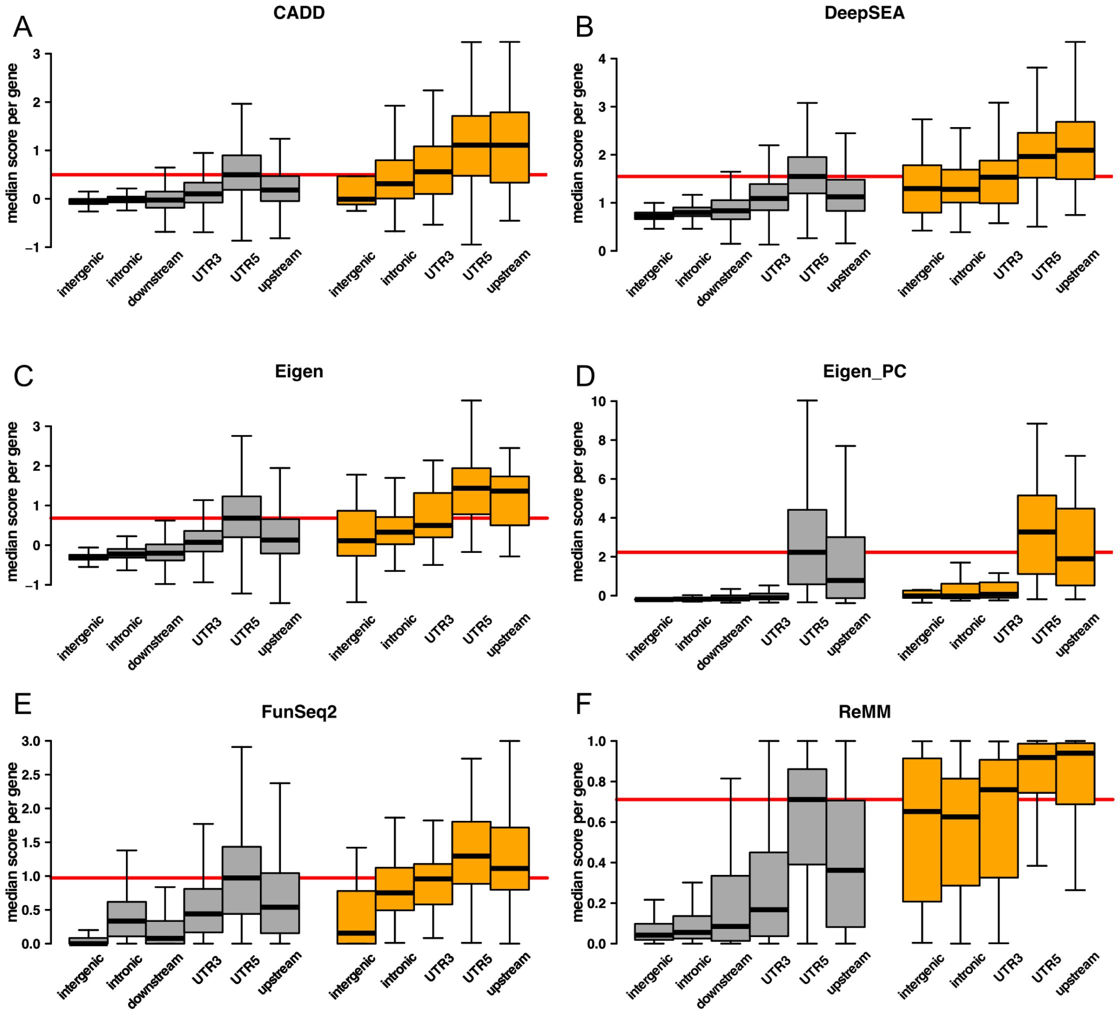

Figure S2. Distribution of pathogenic scores of non-coding SNVs according to the affected type of genomic region.

Boxplots in the panels show the genome-wide distribution of per-region median scores of all non-coding SNVs associated with a given protein-coding gene. Two sets of non-coding SNVs are represented: n=4'960'178 common SNVs without clinical assertions (collectively associated with 18196 protein-coding genes; in grey) and n=737 high-confidence non-coding pathogenic variants (collectively associated with 282 monogenic Mendelian disease genes; in orange). Six types of genomic regions are depicted: intergenic, intronic, 3'UTR, 5'UTR, upstream and downstream regions of associated genes. The downstream region is however not represented in the case of pathogenic non-coding SNVs due to the low set size. Six scores are represented: **A.** CADD non-coding score (3); **B.** DeepSEA functional significance score (4); **C.** Eigen score (5); **D.** Eigen-PC score (5); **E.** FunSeq2 score (6); and **F.** ReMM score (7). The horizontal red line is depicted in each panel at the median value of the 5'UTR distribution for common non-coding SNVs. Table S3 reports the two-sided Wilcoxon test p-values evaluating the null hypothesis that the median pathogenicity score distribution in 5'UTR for common non-coding SNVs is different from the corresponding distribution for pathogenic variants in the 5 types of genomic regions evaluated, i.e: intergenic, intronic, 3'UTR, 5'UTR and upstream.

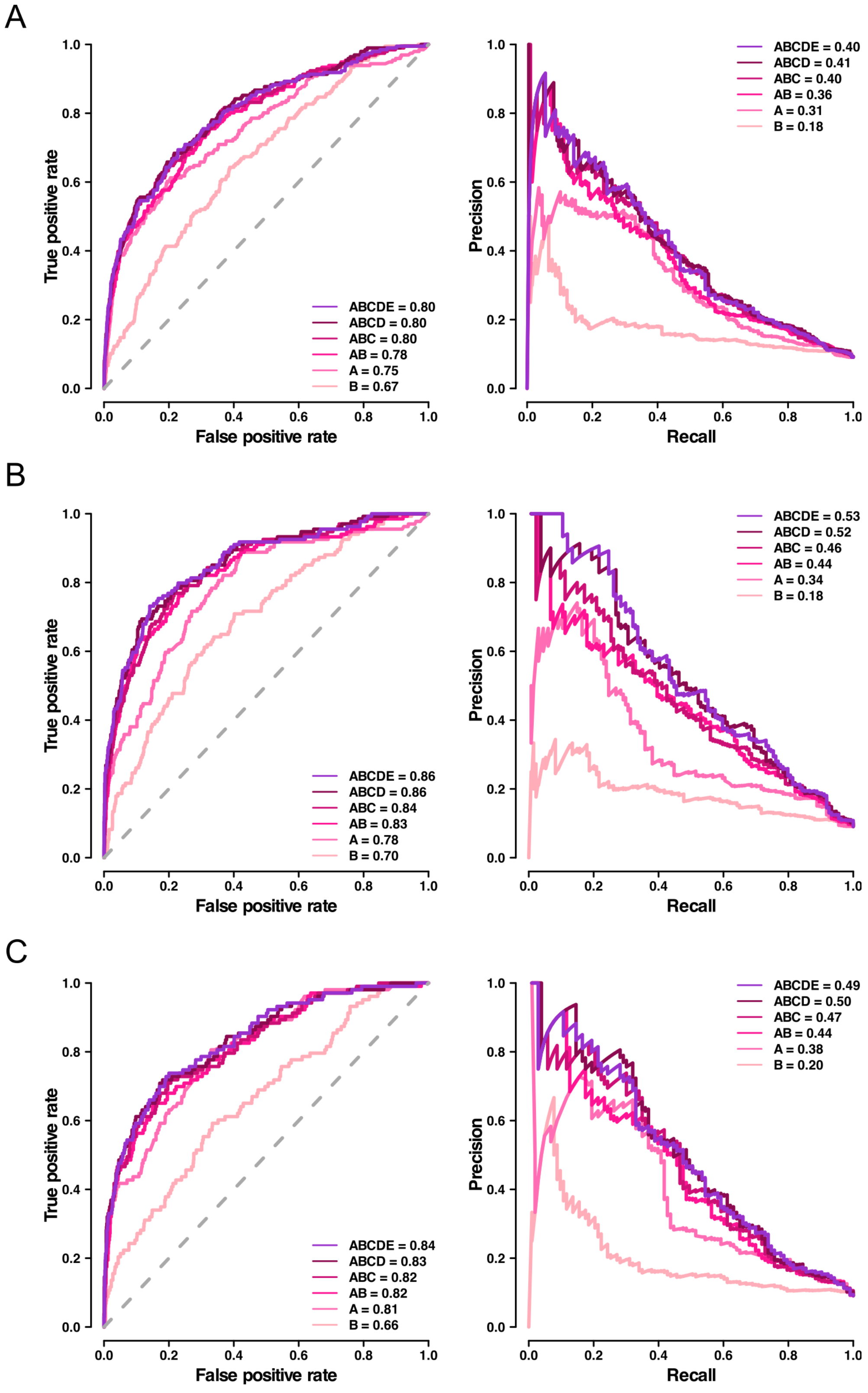

Figure S3. Comparative performance of NCBoost models trained upon different sets of features analyzed independently for each source of pathogenic variants.

The figure represents the area under the receiver operating characteristic curve (AUROC; **Left panels**) and the area under the Precision-Recall curve (AUPRC; **Right panels**) obtained for each of the six feature configurations evaluated (feature categories A, B, A+B, A+B+C, A+B+C+D and A+B+C+D+E) when trained and tested mimicking a ten-fold cross-validation on high-confidence pathogenic non-coding SNVs from the HGMD-DM (**Panel A**), ClinVar (**Panel B**) and Smedley'2016 (**Panel C**) sets.

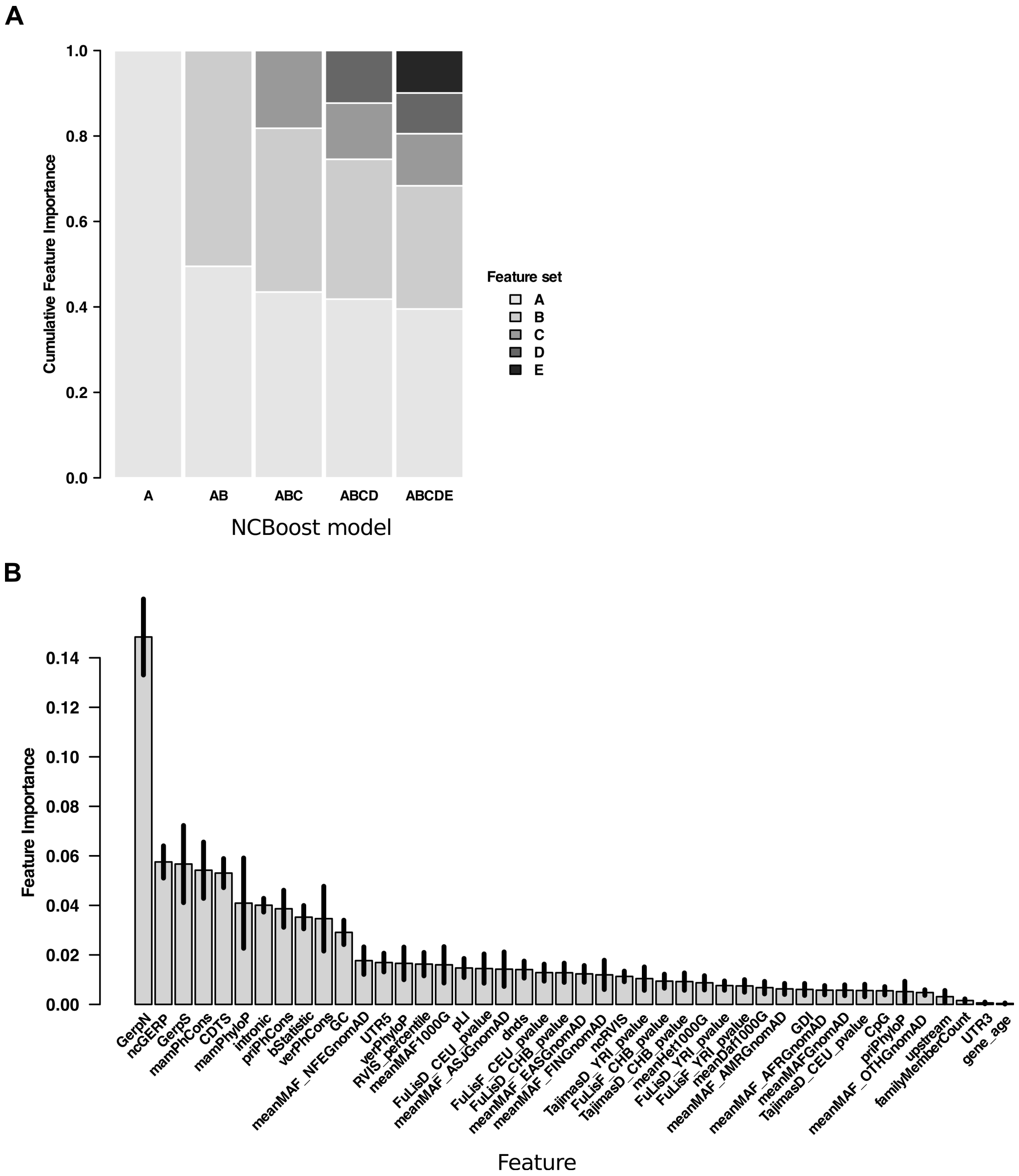

Figure S4. Features importance analysis of NCBoost.
The feature importance represents the average improvement in accuracy brought by a feature to the regression tree branches it is on. A higher value of this metric when compared to another feature implies it is more important for generating an accurate prediction. **A.** Cumulative feature importance of the feature categories A-E under the six NCBoost feature configurations (A, B, A+B, A+B+C, A+B+C+D and A+B+C+D+E) when trained on n=283 high-confidence pathogenic non-coding SNVs and n=2830 common variants without clinical assertions corresponding to the model performances represented in Figure 3. Cumulative importance values were averaged across the 10 independently trained models within the NCBoost bundle, consecutively excluding in each of them 1 of the 10 genome partitions as described in Methods. **B**. Mean and standard deviation of individual feature importance values across the 10 independently trained models within the NCBoost bundle (feature configuration ABCD), consecutively excluding in each of them 1 of the 10 genome partitions (see Methods).

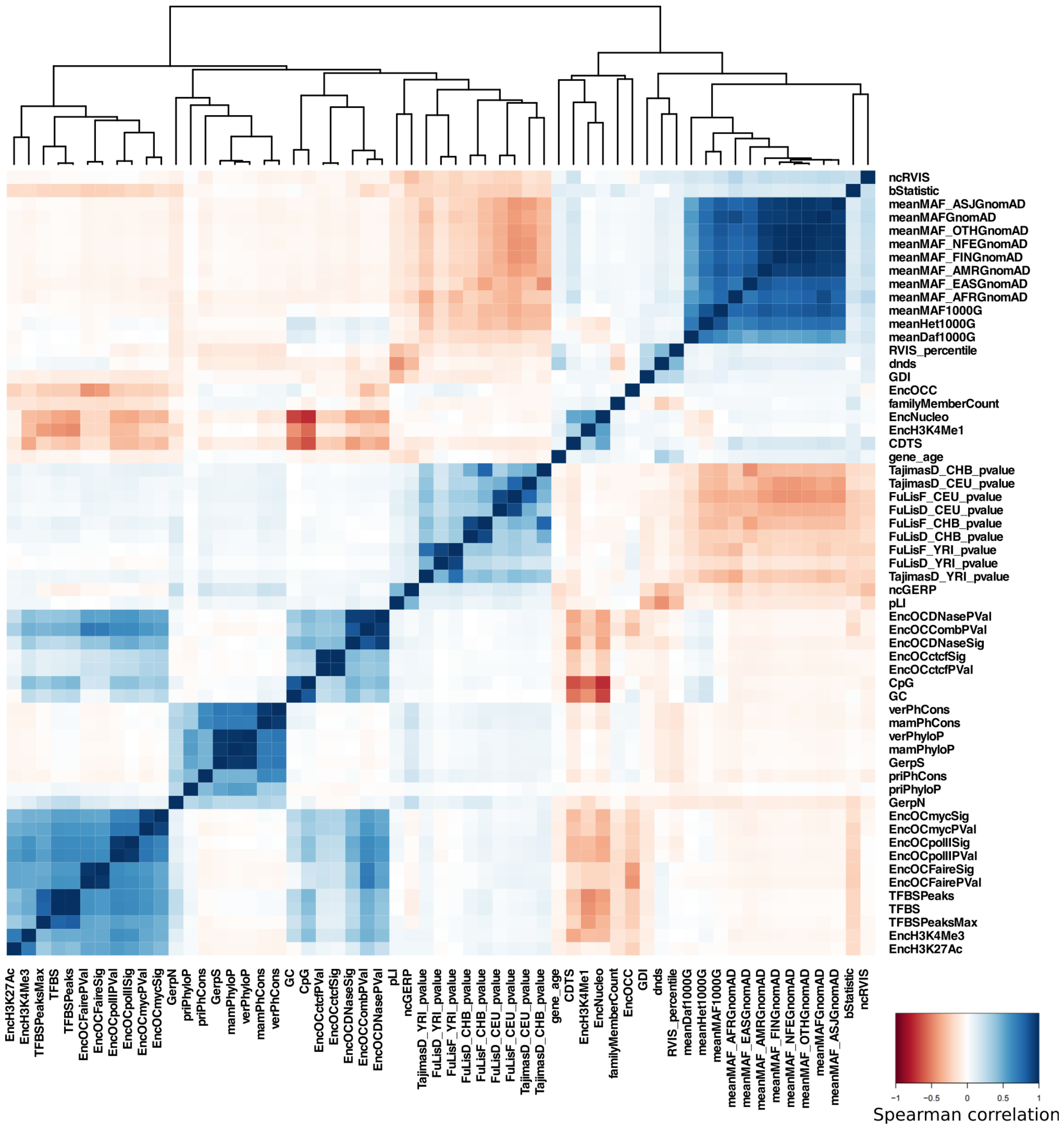

Figure S5. Correlation structure among the features mined in the work.

The figure shows the heatmap representation and associated hierarchical clustering of features based on their Spearman correlation values across the a set of SNVs composed of n=737 non-coding pathogenic variants associated with monogenic mendelian disease genes and n=7370 random common variants.

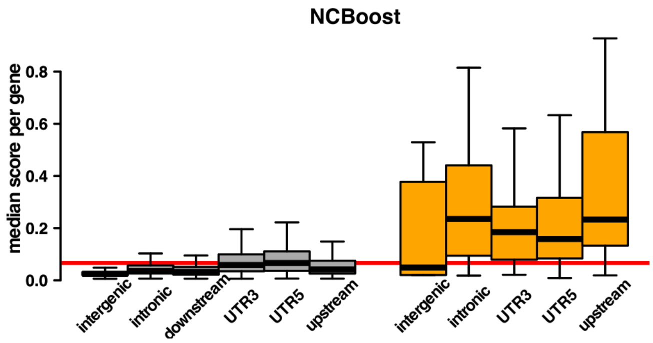

Figure S6. Distribution of NCBoost scores of non-coding SNVs according to the affected type of genomic region.

The figure represents the analogous distributions represented in Figure S2, this time for NCBoost scores**.** Boxplots in the panels show the genome-wide distribution of per-region median pathogenic scores of all non-coding SNVs associated with a given protein-coding gene. Two sets of non-coding SNVs are represented: n=4’960’178 common SNVs without clinical assertions (collectively associated with 18196 protein-coding genes; in grey) and n=737 high-confidence non-coding pathogenic variants (collectively associated with 282 monogenic Mendelian disease genes; in orange). Six types of genomic regions are depicted: intergenic, intronic, 3’UTR, 5’UTR, upstream and downstream regions of associated genes. The downstream region is however not represented in the case of pathogenic non-coding SNVs due to the low set size. Table S3 reports the two-sided Wilcoxon test p-values evaluating the null hypothesis that the median pathogenicity score distribution in 5’UTR for common non-coding SNVs is different from the corresponding distribution for pathogenic variants in the 5 types of genomic regions evaluated, i.e: intergenic, intronic, 3’UTR, 5’UTR and upstream.

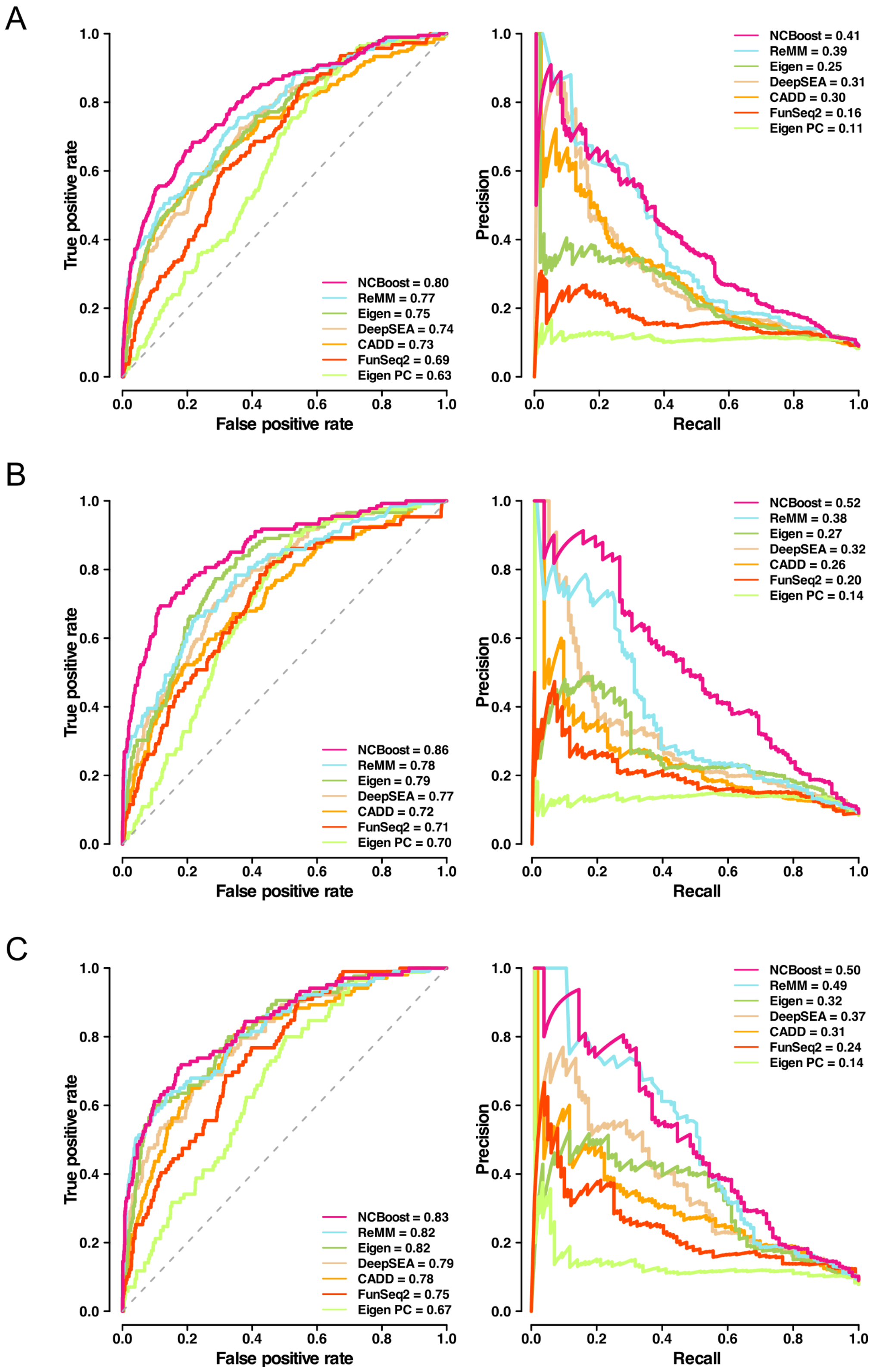

Figure S7. Comparative performance of NCBoost against state-of-the-art methods analyzed independently for each source of pathogenic variants.
Figure shows the the area under AUROC (**Left panels**) and the AUPRC (**Right panels**) obtained for NCBoost (feature configuration ABCD) together with 6 state-of-the-art methods (CADD, DeepSEA, Eigen, Eigen-PC, FunSeq2 and ReMM; see Methods) when tested on high-confidence pathogenic non-coding SNVs from the HGMD-DM set (**Panel A**), ClinVar (**Panel B**) and Smedley'2016 (**Panel C**). The NCBoost model used in each panel as well as the corresponding 'positive' and 'negative' variants correspond to those described in Figure 4 for analogous panels.

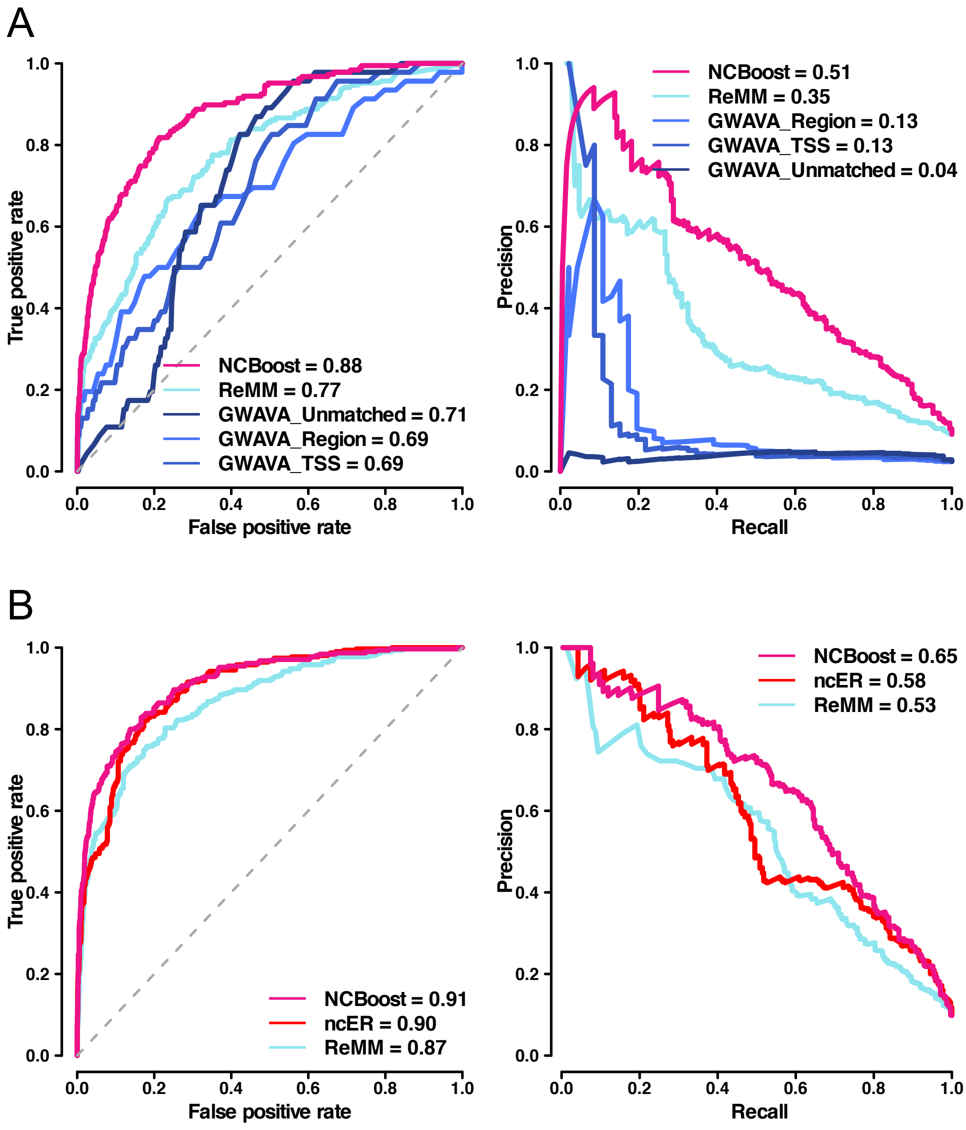

Figure S8. Comparative performance of NCBoost against reference supervised methods trained on pathogenic variants highly enriched in Mendelian diseases.

Figure shows the area under AUROC (**Left panels**) and the AUPRC (**Right panels**) obtained for NCBoost (feature configuration ABCD) together with reference methods based on supervised learning on Mendelian disease variants: ReMM, GWAVA and ncER; see **Methods**) when tested on high-confidence pathogenic non-coding SNVs non overlapping with their training set. **(A).** Benchmark against n=187 high-confidence pathogenic variants curated in this work not overlapping with the HGMD-DM set (**Figure 1**) and the associated 1870 negative variants matched by region (**Methods**). Three versions of GWAVA are reported (‘Unmatched’, ‘TSS’ and ‘Region’, as described in the original publication [1]). **(B).** Benchmark against n=285 high-confidence pathogenic variants curated in this work from Smedley’2016 (**Figure 1**) and the associated 2850 negative variants matched by region (**Methods**).

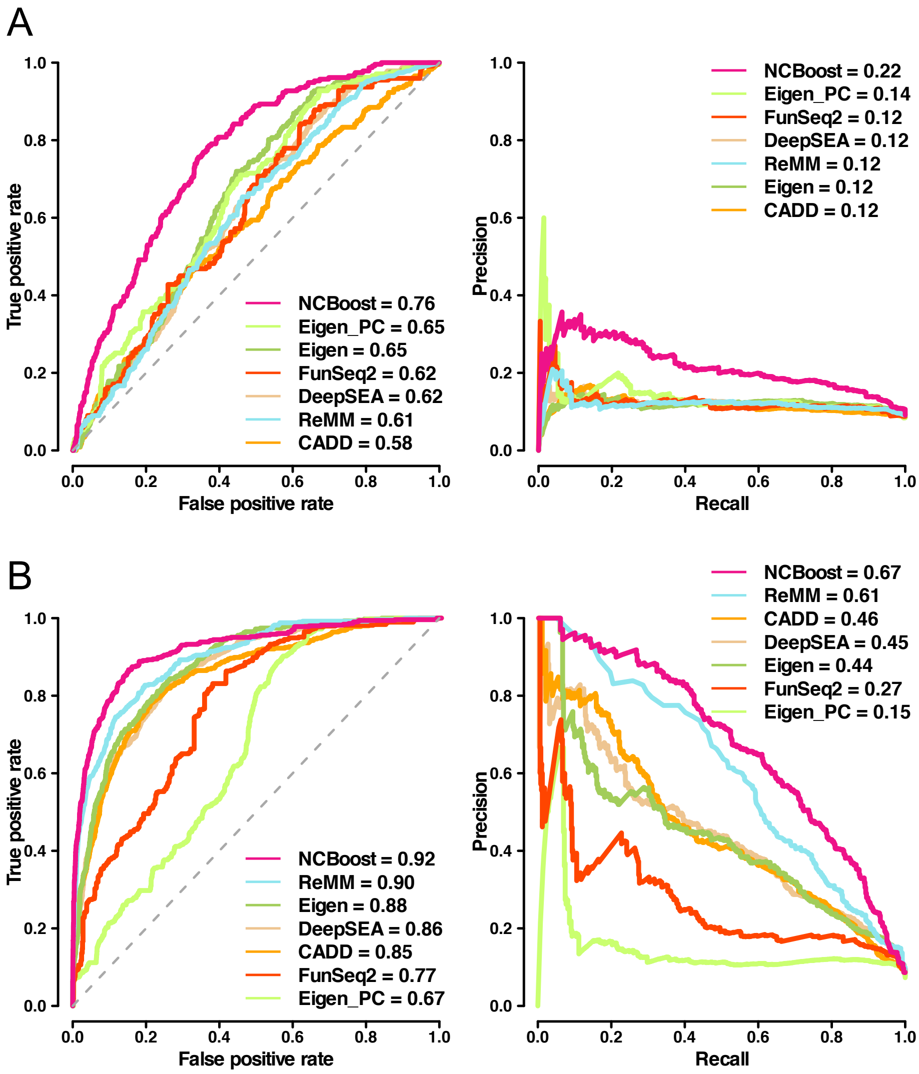

Figure S9. NCBoost capacity to discriminate pathogenic non-coding SNVs seemingly unconstrained in mammals from randomly selected rare variants.

Figure shows the AUROC (**left panels**) and the AUPRC (**right panels**) obtained for NCBoost (configuration of features ABCD) together with 6 state-of-the-art methods (CADD, DeepSEA, Eigen, Eigen-PC, FunSeq2 and ReMM; see Methods) when tested on the following subsets of the 'positive set' of n=737 high-confident set of pathogenic non-coding SNVs: (**A**) 234 high-confidence pathogenic variants within the initial set seemingly unconstrained in mammals, defined here as those with PhastCons and PhyloP scores for Mammals below the median value of the 7370 common variants sampled for training (0.001 and -0.014, respectively); and (**B**) the 503 variants seemingly constrained in mammals, defined here as those with PhastCons and PhyloP scores above such median values. In both cases we used the associated negative set of common variants matched by region as described in **Methods**. Of note, no selection of negative variants according to PhastCons and PhyloP scores was imposed in either scenario.

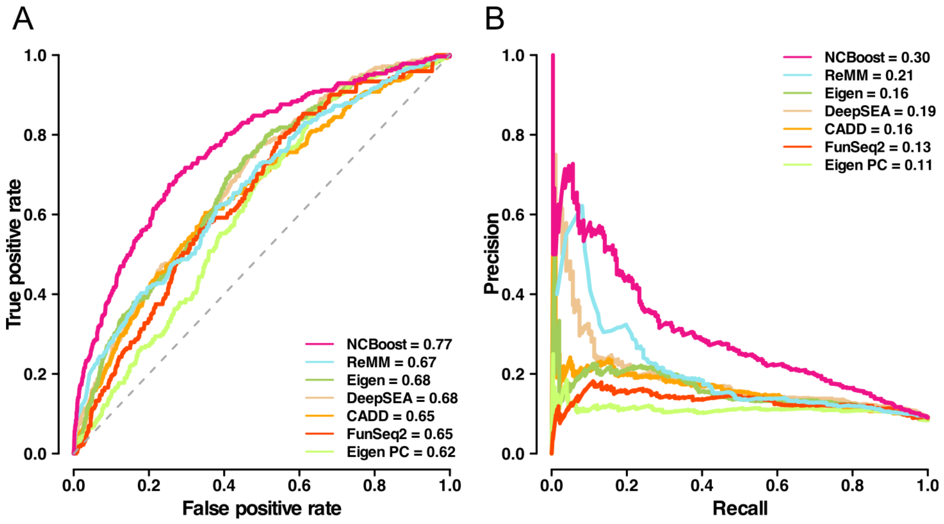

Figure S10. NCBoost capacity to discriminate pathogenic non-coding SNVs from randomly selected rare variants.

Figure shows the AUROC (**Panel A**) and the AUPRC (**Panel B**) obtained for NCBoost (configuration of features ABCD) together with 6 state-of-the-art methods (CADD, DeepSEA, Eigen, Eigen-PC, FunSeq2 and ReMM; see Methods) when tested on the same 'positive set' of n=283 high-confident set of pathogenic non-coding SNVs as in Figure 4 and on a negative set that -rather than of common variants- is composed of 2830 randomly selected rare variants (allele frequency < 1%) matched by region. We note that no re-training of NCBoost was done here but used the same NCBoost $_{ABCD}$ model trained as described for Figure 3 and Figure 4.

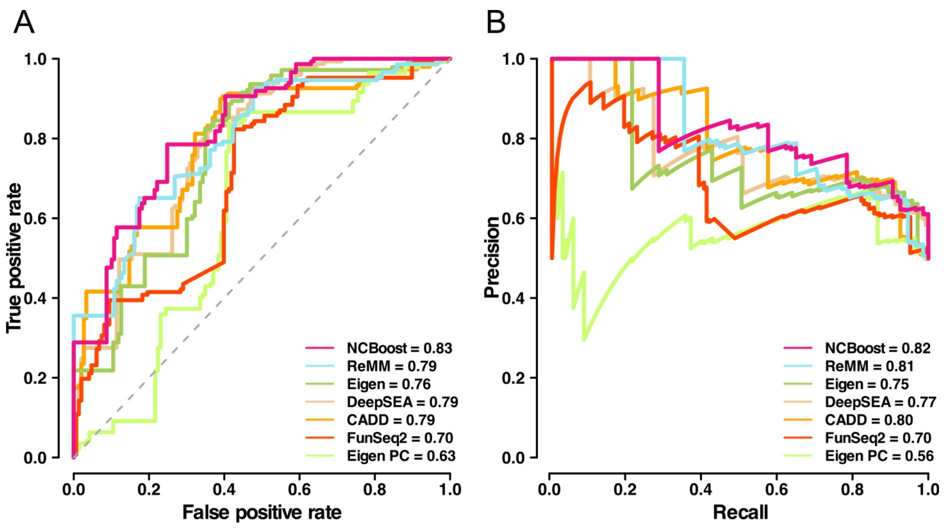

Figure S11. NCBoost capacity to discriminate pathogenic and non-pathogenic variants within the same non-coding region of a given gene.
Figure shows the AUROC (**Panel A**) and the AUPRC (**Panel B**) obtained for NCBoost (configuration of features ABCD) together with 6 state-of-the-art methods (CADD, DeepSEA, Eigen, Eigen-PC, FunSeq2 and ReMM; see Methods) when tested on a set of 149 region-matched pairs of pathogenic and random common variants associated with 54 unique genes. We note that no re-training of NCBoost was done here but used the same NCBoost $_{ABCD}$ model trained as described for Figure 3 and Figure 4.

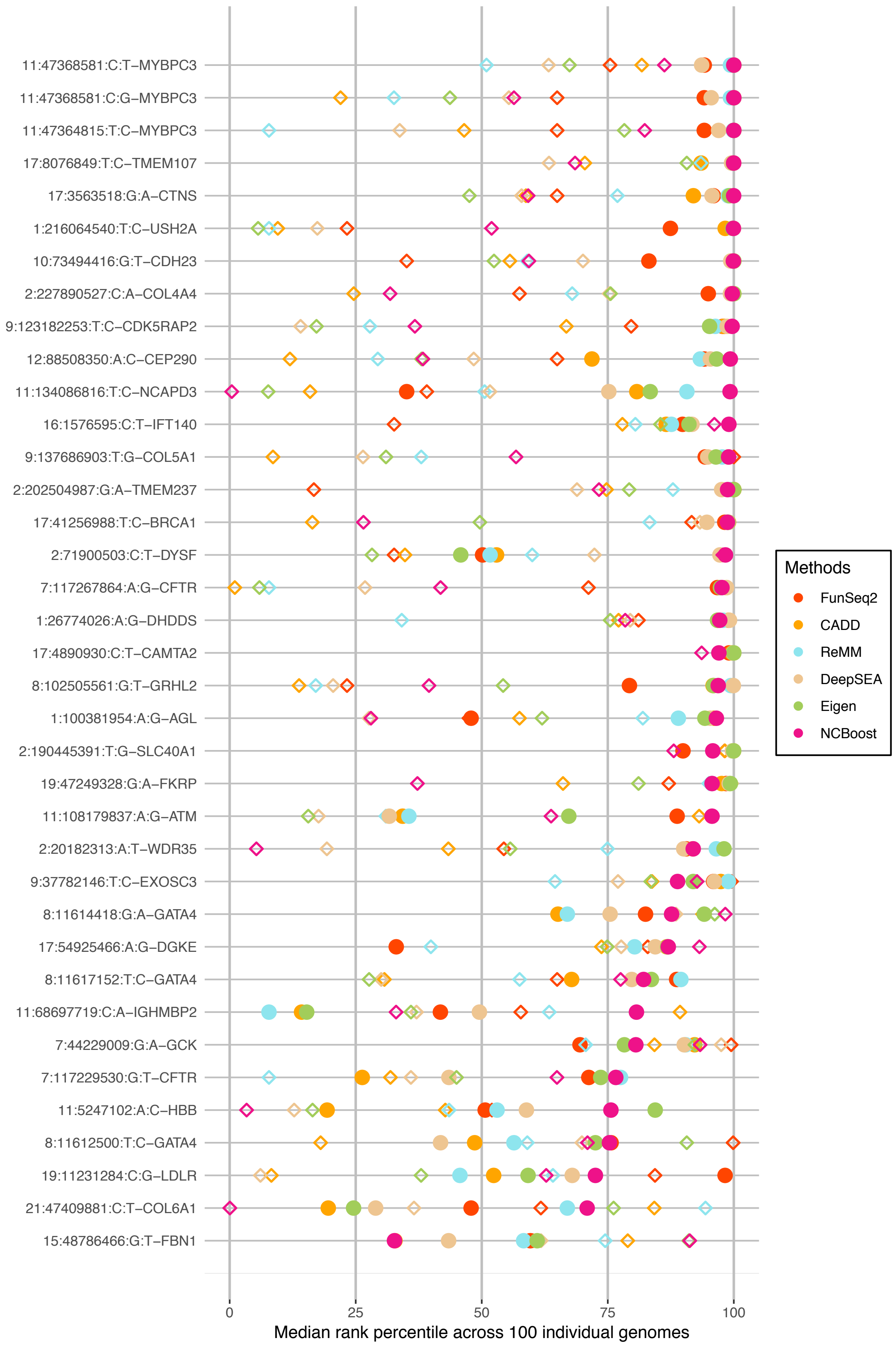

Fig. S12. Prioritization of 37 recently reported non-coding pathogenic variants in autosomal chromosomes and its associated 37 internal control negative common variants within simulated disease genomes. The median across the 100 simulated disease genomes of the within-individual rank percentile of a variant (x-axis) is shown for each method evaluated (following the color code indicated in the legend) for each of the 37 recently reported pathogenic SNVs evaluated (solid circles, one line per pathogenic variant as listed in the y-axis) together with the associated common variant used as internal control (diamond symbols). The genomic position and associated gene of the pathogenic variant is indicated. Complete details are provided in **Additional File 5: Supplementary Table S5**.

Supplementary References

1- Dang VT, Kassahn KS, Marcos AE, Ragan MA. Identification of human haploinsufficient genes and their genomic proximity to segmental duplications. Eur J Hum Genet. 2008;16(11):1350–7.

2- Lek M, Karczewski KJ, Minikel E V., Samocha KE, Banks E, Fennell T, et al. Analysis of protein-coding genetic variation in 60,706 humans. Nature. 2016;536(7616):285-91. http://www.nature.com/doifinder/10.1038/nature19057.

3- Kircher M, Witten DM, Jain P, O'roak BJ, Cooper GM, Shendure J. A general framework for estimating the relative pathogenicity of human genetic variants. Nat Genet . 2014;46(3):310–5. http://dx.doi.org/10.1038/ng.2892

4- Zhou J, Troyanskaya OG. DeepSEA Predicting effects of noncoding variants with deep learning–based sequence model. Nat Methods. 2015;12(10):931–4. http://www.nature.com/doifinder/10.1038/nmeth.3547

5- Ionita-Laza I, McCallum K, Xu B, Buxbaum JD. A spectral approach integrating functional genomic annotations for coding and noncoding variants. Nat Genet. 2016;48(2):214–20. http://www.nature.com/doifinder/10.1038/ng.3477

6- Fu Y, Liu Z, Lou S, Bedford J, Mu XJ, Yip KY, et al. FunSeq2: a framework for prioritizing noncoding regulatory variants in cancer. Genome Biol. 2014;15(10):480. http://genomebiology.biomedcentral.com/articles/10.1186/s13059-014-0480-5

7- Smedley D, Schubach M, Jacobsen JOOB, Köhler S, Zemojtel T, Spielmann M, et al. A Whole-Genome Analysis Framework for Effective Identification of Pathogenic Regulatory Variants in Mendelian Disease. Am J Hum Genet. 2016;99(3):595–606.
